# Exploring AI in metasurface structures with forward and inverse design

**DOI:** 10.1016/j.isci.2025.111995

**Published:** 2025-02-15

**Authors:** Guantai Yang, Qingxiong Xiao, Zhilin Zhang, Zhe Yu, Xiaoxu Wang, Qianbo Lu

**Affiliations:** 1 Frontiers Science Center for Flexible Electronics (FSCFE) Institute of Flexible Electronics (IFE), Northwestern Polytechnical University, Xi’an 710072, China; 2 School of Automation, Northwestern Polytechnical University, 127 West Youyi Road, Beilin District, Xi’an 710072, China; 3 College of Optical Science and Engineering, Zhejiang University, Hangzhou 310027, China

**Keywords:** Optics, Artificial intelligence, Materials science

## Abstract

As an artificially manufactured planar device, a metasurface structure can produce unusual electromagnetic responses by harnessing four basic characteristics of the light wave. Traditional design processes rely on numerical algorithms combined with parameter optimization. However, such methods are often time-consuming and struggle to match actual responses. This paper aims to give a unique perspective to classify the artificial intelligence(AI)-enabled design, dividing it into forward and inverse designs according to the mapping relationship between variables and performance. Forward designs are driven by intelligent algorithms; neural networks are one of the principal ways to realize reverse design. This paper reviews recent progress in AI-enabled metasurface design, examining the principles, advantages, and potential applications. A rich content and detailed comparison can help build a holistic understanding of metasurface design. Moreover, the authors believe that this systematic and detailed review will pave the way for future research and the selection of practical applications.

## Graphical abstract

**Figure:**
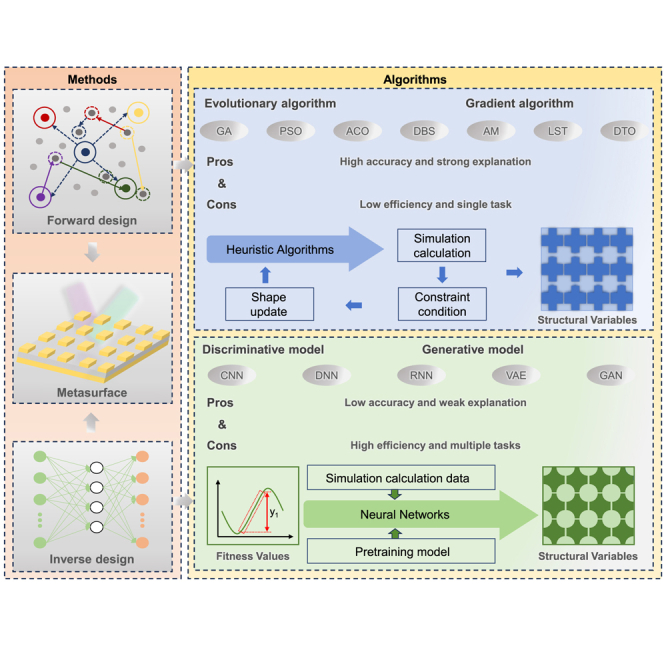

## Introduction

Metasurfaces^1^^,^^2^^,^^3^ are ultrathin array nanostructures whose optical response can be engineered by designing periodic array structures or materials. As artificial planar devices, metasurfaces can generate unusual electromagnetic responses using the amplitude, phase, polarization, and frequency of light waves. Recent applications of metasurfaces have expanded rapidly in several fields. For example, in optical information processing, metasurfaces bring transformative enhancements to virtual reality devices and artificial intelligence (AI) hardware by aiding hologram generation and optical acceleration. Smart metasurfaces^4^^,^^5^^,^^6^ are used for control mode design, beamforming, and spectrum multiplexing to support next-generation wireless communication networks, such as 6G. In addition, metasurfaces also show enormous potential in optical sensing,^7^ optical imaging,^8^ beam segmentation,^9^ optical filters,^10^ and microlenses.^11^ In designing metasurface structures, multiple full-wave electrodynamic simulations have been performed to optimize the desired optical response or function.^12^ Various brute force simulation methods based on electromagnetic (EM) Maxwell equation solvers have been used to design and optimize nanostructures.^13^ However, in these traditional metasurface design processes, due to the high complexity of metasurface design and increased regulatory parameters, the design cost is high, the design time is long, and many experienced researchers must be involved. For the inverse problem of nanounit structures with specific EM responses, researchers can only empirically invert simple optical responses due to multiple locally optimal solutions, which has greatly limited metasurface development. Research on intelligent metasurfaces is appearing, and its function is dynamically regulated by the design of the control mode. In addition, with the continued development of related research, hypersurface design methods combining optimization algorithms and neural networks have emerged.

In general, all examples require an objective function match, which is the definition of reverse design. AI for forward design generally means using AI as a Maxwell equation solver to speed up the simulation. The forward design we define is based on parameter-to-response mapping; algorithms are designed to efficiently search the large design space, and the design process is usually guided by evolutionary algorithms^14^ (e.g., heuristic or gradient-based methods). These algorithms can discover non-intuitive, irregularly shaped photonic structures that outperform empirically based designs in many applications, including metasurfaces, photonic crystals, and silicon photonic elements.^15^^,^^16^ Fundamentally, these algorithms are rule-based approaches that fine-tune the search strategy by optimizing the algorithm’s search parameter space to find the optimal design parameters, involving iterative search steps on a case-by-case basis, often relying on numerical simulations of each step to generate intermediate results. However, the cost functions of many optimization algorithms are non-convex, meaning that they have many local minimums and saddle points, which cannot guarantee global optimality in a complex environment. For complex devices with non-intuitive shapes and high-dimensional problems, blind simulation iteration is very resource-consuming and can usually optimize only one task. In addition, regularizing the weight parameters is very important in the optimization process: the risk of over-regularization is high, and there is a contradiction between the optimization task’s complexity and the model’s simplification. This stochastic algorithm is limited by its inherent random search characteristics, which makes it inadequate in complex designs involving multi-constraint problems.

Inverse design has a much longer history than neural networks. Many optimization methods, such as adjoint methods, topology optimization, and genetic algorithms (GAs), have been used for the inverse design of hypersurfaces. The inverse design we define is based on a response to parameter mapping; the design goal is not limited to the specific index of a particular feature, and the algorithm has a specific generalization ability. A pre-trained model based on a neural network is used to predict the unknown structural parameters corresponding to the target response. Neural networks learn complex correlations or mappings between inputs and outputs with minimal human intervention.^17^^,^^18^ A neural network consists of several hidden layers of multiple neurons trained on generalizable datasets. Its unique strength lies in its data-driven approach, which allows models to automatically discover useful information from large amounts of data, unlike physical or rule-based approaches. Neural network-based discriminant models^19^^,^^20^ and generative models^21^ have been used for inverse design, where the discriminant models include convolutional neural networks (CNNs), deep neural networks (DNNs), and recurrent neural networks (RNNs). Generative models include variational autoencoders (VAEs) and generative adversarial networks (GANs). Although these show that neural networks can predict the structure of metasurfaces when processing a large number of EM simulation datasets simultaneously, the inverse design process using neural networks still faces the problem of difficult convergence. Chiefly due to the task of metasurface design, the mapping relationship between performance and design is often many-to-many; that is, multiple design parameters may correspond to similar target ones. This complex nonlinear relationship brings many challenges to inverse design. For example, in many-to-many mapping, the neural network model may be unable to learn all potential design solution sets effectively, resulting in an optimization bias toward some local solutions and failing to fully explore the design space. Even if the network can generate designs that meet the target performance, its output design schemes may be highly similar in form or structure and lack diversity, thus limiting the flexibility of the design. When the design task or goal changes, the model may no longer be applicable, requiring retraining or fine-tuning. In the past few years, neural networks have been successfully applied in nanophotonics for device optimization^22^^,^^23^^,^^24^^,^^25^ and inverse design^26^^,^^27^^,^^28^ to obtain the desired optical response and directional function.

This paper proposes classifying metasurface structural design methods empowered by AI algorithms: forward and inverse design. The main difference lies in the mapping relationship between the structural parameters and the response and the process of obtaining the target structural parameters. Based on the mapping relationship between structural parameters and responses, we define optimization and evolutionary algorithms that map structures to responses as forward design and neural network algorithms that directly derive structural parameters from responses as inverse design. We introduce the background of AI-empowered metasurface design methods, highlighting their formation, development, and advantages. Next, we discuss several major algorithmic structures, ranging from basic heuristic and gradient algorithms to advanced neural networks and hybrid models incorporating other optimization methods, emphasizing their potential in designing metasurfaces, photonic crystals, plasmonic nanostructures, and integrated silicon photonic devices. These algorithms can search for map design parameters—such as geometry, materials, topology, and spatial arrangements—to achieve both forward and inverse design. Finally, we address the challenges and prospects of AI-based metasurface structural design. We believe that the comprehensive content and detailed comparisons can offer a holistic understanding of metasurface design.

## Overview of metasurface forward design and inverse design

**Figure 1:**
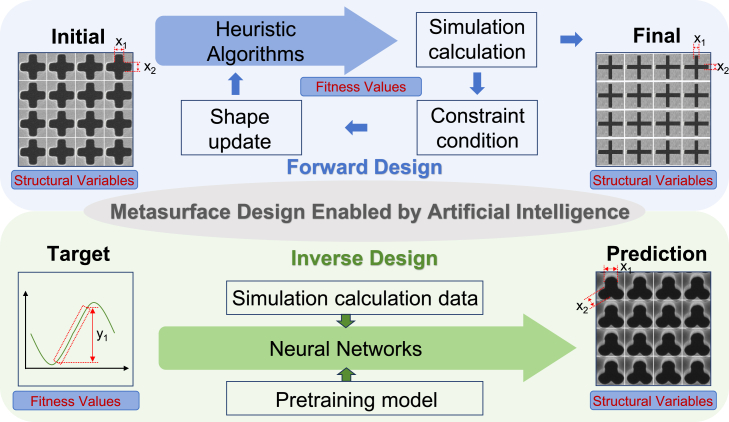
The flow chart of forward design and inverse design

In this article, we propose distinguishing between the forward and inverse design of metasurface structures by defining the mapping relationships between variables and performance. Based on this mapping relationship, we define design methods that map from variables to performance as the forward design of metasurface structures and methods that map from performance to variables as the inverse design of metasurface structures, as shown in Figure 1. We believe that the primary distinction between forward and inverse design lies in configuring input and output parameters within the mapping relationship. Specifically, forward design methods, such as heuristic and gradient-based intelligent optimization algorithms, involve adding physical constraints to accelerate the optimization of target metrics. Therefore, heuristic and gradient-based intelligent optimization algorithms fall under forward design for metasurface structures. Conversely, the mechanism of inverse design using algorithms such as neural networks involves training network models with large datasets, in addition to leveraging the prior knowledge of the dataset and the sophisticated learning capabilities of complex network structures to uncover the underlying rules from performance to variables, thus achieving a design entirely based on performance-to-variable mapping. The key difference between inverse and forward design is that inverse design lacks direct physical interpretability. Unlike forward design, which directly applies traditional physical formulas for derivation, inverse design relies on extensive datasets to learn the underlying rules of the mapping relationship.

**Figure 2:**
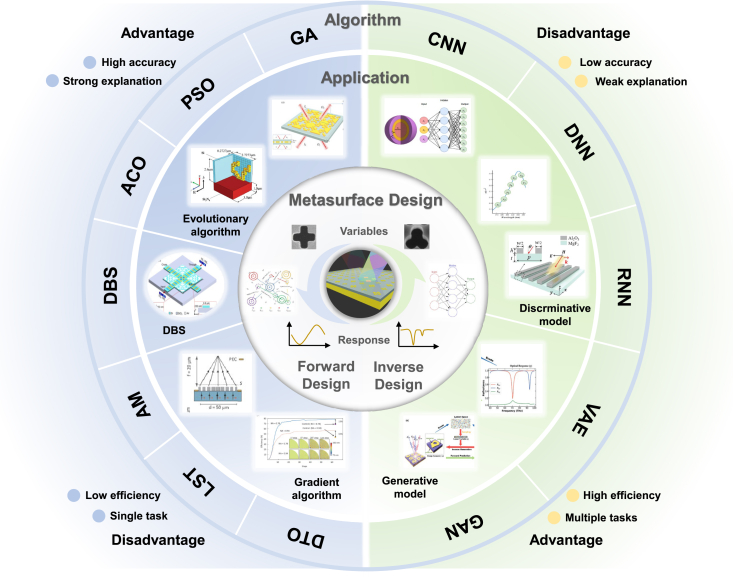
A dial illustration of AI-based metasurface structural design methodology The outer section lists a specific set of algorithms and the pros and cons of forward design and reverse design. The middle ring represents the spectral response curve and the categories of forward design and reverse design methods. The interior area depicts the design of the metasurface structure based on forward design and reverse design development. Reproduced with permission, from Luo et al.^33^ Copyright 2022, Optical Society of America. Reproduced with permission, from Zhu et al.^34^ Copyright 2019, American Chemical Society. Reproduced with permission, from Liu et al.^35^ Copyright 2024, IEEE. Reproduced with permission, from Mansouree et al.^36^ Copyright 2021, American Chemical Society. Reproduced with permission, from Ma et al.^37^ Copyright 2019, Wiley Online Library. Reproduced with permission, from Peurifoy et al.^38^ Copyright 2018, AAAS.

The intelligent optimization algorithms for forward design of metasurface structures include heuristic algorithms,^29^ direct binary search (DBS) algorithms, gradient optimization algorithms, and hybrid algorithms incorporating neural networks, as shown in Figure 2. Notably, heuristic algorithms encompass GAs,^30^ particle swarm optimization (PSO),^31^ and ant colony optimization (ACO).^32^ Gradient optimization algorithms include adjoint method (AM), level set optimization (LST), and density topology optimization (DTO). These optimization strategies are based on electromagnetic numerical computations, where electromagnetic simulation tools solve design problems by discretizing Maxwell’s equations in the frequency and time domains. By setting sufficient grid resolution and iteration steps, the optical characteristics of a given structure can be accurately calculated. Intelligent algorithms serve as the guiding framework for the forward design process. However, their limitations include non-convexity, high dimensionality, and over-regularization. The cost functions of many optimization algorithms have been proven to be non-convex, meaning they have many local minimums and saddle points, which cannot guarantee a global optimum in a complex environment. For complex devices with non-intuitive shapes and high-dimensional problems, blind simulation iteration is very resource-consuming, and usually, only one task can be optimized. In addition, regularizing weight parameters is particularly important in the optimization process. The risk of over-regularizing is high, and the contradiction between the optimization task’s complexity and the model’s simplification still exists.

Neural network inverse design models for metasurface structures include generative and discriminative models(Figure 2).^39^ Generative models include CNNs, DNNs, and RNNs, while discriminative models include VAEs and GANs.^40^^,^^41^ Hybrid models that combine various structures are also used in the design. Neural network models capture the complex mappings between design space and response space effectively. This data-driven approach has propelled research in metasurface structural design in predicting responses and inversely predicting variables.^42^ Currently, neural networks are the best choice for solving inverse design problems of complex geometries. Unlike traditional parameter tuning, neural network-based algorithms exhibit inherent advantages in solving efficiency and diverse sample outputs. However, there are still some limitations, and the lack of high-quality datasets limits the training of neural networks because neural network model training is based on datasets. The unclear or excessively complex feature extraction, the high similarity between different data, and the many-to-one mapping between response and structural parameters can all affect weight updates in model training, resulting in underfitting or overfitting and inaccurate model prediction. Moreover, the algorithms proposed so far can usually only solve reverse design problems with limited degrees of freedom and are limited to a specific structure.^43^ For multiband metasurface-based designs, there is still considerable room to meet multiple functional objectives at the same time (such as reflection, transmission, and absorption properties of different bands) and address multiple design constraints, such as physical limitations and manufacturing feasibility. In multiband design, the performance requirements of each band increase the complexity of design parameters, resulting in a rapid expansion of the search space and a higher design space dimensionality. The functional requirements of different bands may conflict, and more functions mean a more complex structure, so the design needs to consider many factors, such as electromagnetic performance constraints, material limitations, and manufacturing processes. Moreover, structural inverse design based on neural networks is not easily physically interpretable, and it is still difficult to explore new dimensions in terms of multi-dimensional space optimization and multi-text output, which requires additional simulation time to retrain the network.

## Forward design of metasurface

Based on the mapping relationship from variables to performance, metasurface structural design using intelligent optimization algorithms is defined as forward design. Intelligent optimization algorithms enhance the efficiency of traditional metasurface structural design by adding physical constraints to accelerate the optimization of design metrics. These algorithms include heuristic algorithms, DBS, and gradient optimization algorithms. By simulating the evolutionary and collective behaviors observed in nature, these algorithms iteratively refine design parameters to optimize the performance of complex systems. Heuristic algorithms like GAs generate new design solutions through selection, crossover, and mutation operations, making them suitable for global optimization problems. PSO achieves rapid convergence through collaboration and learning among particles, which is particularly effective for continuous optimization problems. ACO, which simulates the foraging behavior of ants, excels in global search tasks. Gradient optimization algorithms, such as AM and LST, perform precise optimization by calculating the gradient information of the objective function. AM simplifies gradient calculations through adjoint equations, making it suitable for optimization in high-dimensional design spaces. LST improves overall performance by locally modifying metasurface unit structures. Intelligent forward-design algorithms are commonly used in high-dimensional complex designs, such as optical metasurfaces and acoustic devices, where intelligent algorithms effectively enhance design efficiency. Compared to traditional design methods, this approach provides more diverse search strategies to achieve high-performance device structures more efficiently.^29^

The performance index of the forward-design algorithm is evaluated from several aspects: parameter freedom, applicable data type, accuracy, and efficiency. Table S1 shows the performance indicators comparing different forward-design algorithms, dividing performance into five levels. These indexes reflect the advantages and disadvantages of these algorithms in metasurface design so that reasonable choices can be made based on such factors as calculation cost and accuracy. Table S3 shows the computational requirements of the algorithms, including analyzing requirements for large-scale metasurface design. First, forward design algorithms, such as GA, PSO, ACO, DBS, AM, and DTO, usually need to solve the complexity problem of hypersurface structures in the design stage. The parameter freedom of these algorithms is closely related to the computing resource requirements. Heuristic optimization algorithms such as GA, PSO, and ACO usually require considerable iteration to find the global optimal solution, especially for high-complexity hypersurface structures. The computation cost increases exponentially. These algorithms may require significant computational resources for large-scale metasurface design, especially when multiple calculations or evaluations are required. One gradient design method, DTO, improves calculation efficiency by optimizing design parameters. Although its calculation cost is lower, it depends strongly on gradient calculation, which may require a high-precision numerical solution of the design model. In general, the computation cost of a forward design algorithm is high when dealing with complex design problems, especially when the scale of metasurface design increases, which may require substantial computational resources to ensure accuracy.

### Heuristic algorithm for forward design

#### GA for forward design

##### GA

**Figure 3:**
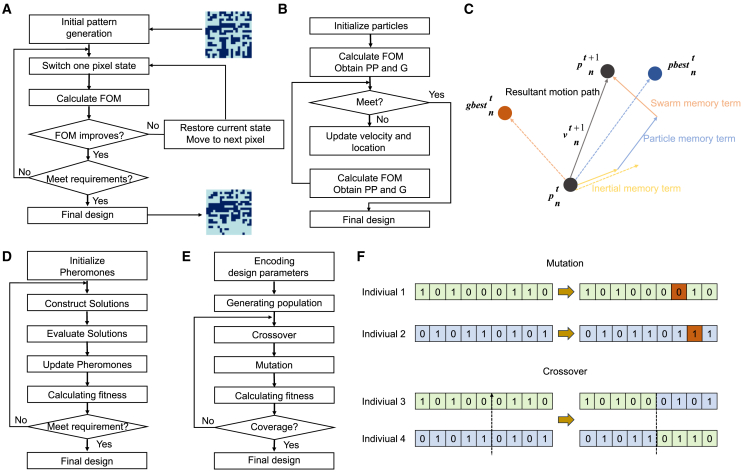
Partial forward design algorithm frame diagram (A) The optimization process of Direct Binary Search. (B) The optimization process of particle swarm optimization. (C) The particle renewal process of particle swarm optimization. (D) The optimization process of ant colony optimization. (E) The optimization process of genetic algorithm. (F) The cross-mutation process of genetic algorithm.

GAs are a typical heuristic method. This global search algorithm mimics the process of natural evolution, specifically the biological rule of survival of the fittest, to search for optimal global solutions. The GA algorithm is widely used because of its effectiveness, simplicity, and intuitiveness.^44^ Heuristic optimization relies on a limited parameterization of the solution space, followed by random testing of a large set of parameters. Figure 3 shows a flowchart of the GA used in this study for the forward design of metasurface structures. Initially, the GA requires encoding the problem parameters and mapping them onto chromosomes rather than using these parameters directly as encoding themselves. A population is then created using an appropriate encoding program, and fitness values are calculated. Through operations such as crossover, selection, and mutation on the population of chromosomes, information on the chromosomes is altered to generate a new population.^45^ (Figures 3E and 3F) The selection operation follows the roulette wheel rule, where the probability of selecting a particular operation is proportional to its fitness value. The crossover operation selects two chromosomes from the current population to mate and produce offspring that are expected to combine their parents’ strengths. The mutation operation flips or changes the value of a randomly selected position for the mutating individual. Mutation introduces new genes, maintains population diversity, and helps avoid local optima. This process is repeated over multiple generations, gradually converging to the optimal solution for the problem. A GA is particularly effective in solving complex problems that require simultaneous improvement of multiple system characteristics. This makes them suitable for real-world problems where a single, well-defined optimal solution may not exist.

##### Application

**Figure 4:**
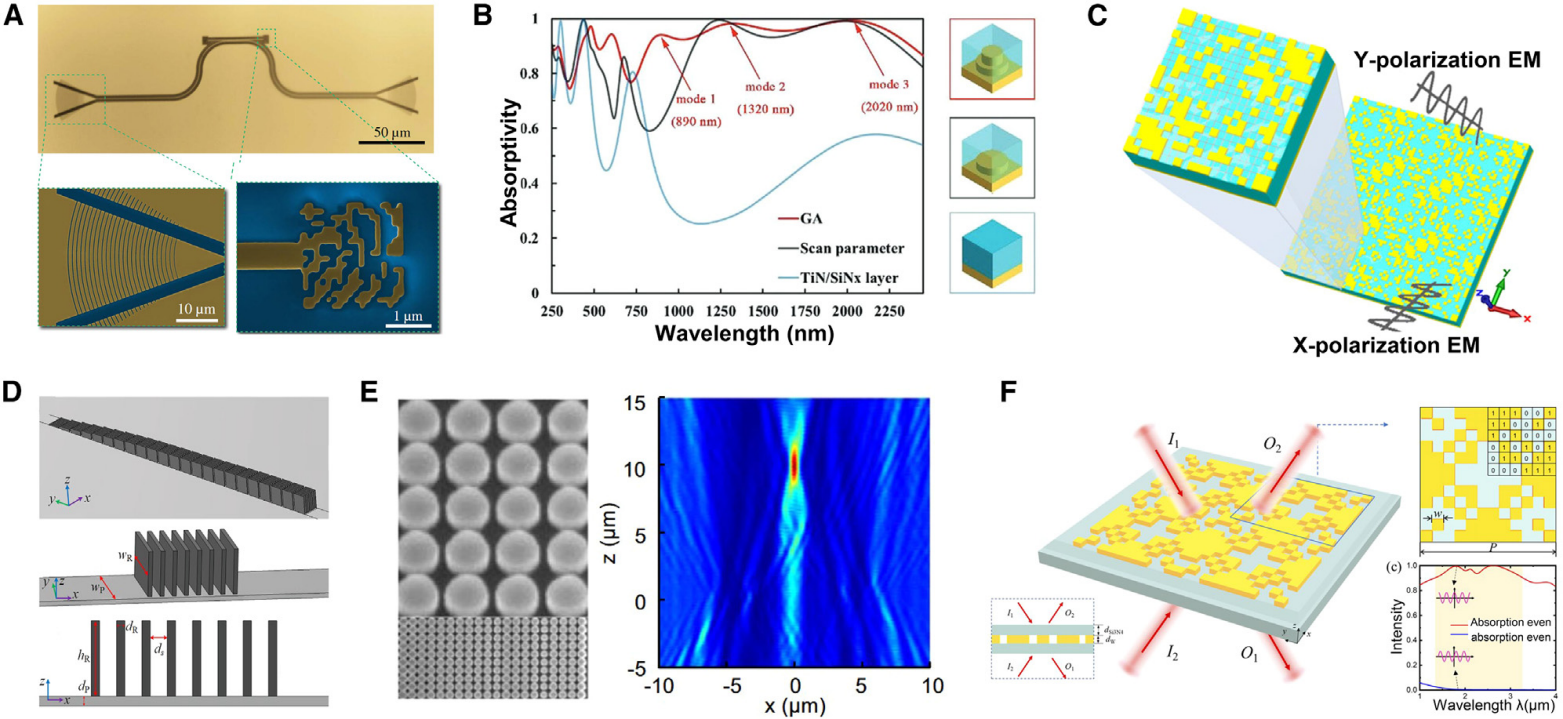
GA adopted for the forward design of metasurface (A) Upper panel: an optical image of the FP cavity. Reproduced with permission, from Li et al. Copyright 2017,^47^ Chinese Laser Press. (B) Simulated absorptive spectrum of the GA-based metasurface. Reproduced with permission, from Jiang et al. Copyright 2021,^48^ Wiley Online Library. (C) Schematic illustration of the optimized metasurface based on Topology-1. Reproduced with permission, from Sui et al. Copyright 2016,^49^ American Institute of Physics. (D) Schematic 3D model of the pillared elastic metasurface. Reproduced with permission, from Xu et al. Copyright 2023,^50^ Elsevier. (E) Scanning electron microscopy images of the meta-atoms arrangement for lenses with NA = 0.51 and 0.77. Reproduced with permission, from Cai et al. Copyright 2020,^51^ Springer Nature. (F) Schematic diagram of the proposed tungsten-based CPA metamaterial absorber. Reproduced with permission, from Luo et al. Copyright 2022,^33^ Optical Society of America.

GAs are widely recognized for their effectiveness in global optimization problems, making them particularly suitable for the design of metasurfaces. GAs accommodate both discrete and continuous optimization, enabling diverse design applications. As shown in Figure 4, Yu et al. utilized GA for optimizing complex amplitude metasurface antenna arrays, realizing cosecant-squared beams with numerical and experimental validation through a 10-unit line array.^30^ Liu et al. applied a GA-driven collaborative approach to create metamaterials with tailored microwave reflectance and infrared emissivity, achieving consistent results across simulations, calculations, and experiments.^46^

Further, Yu et al. employed GAs for optimizing on-chip broadband reflectors and Fabry-Perot cavities on silicon-on-insulator films, achieving over 97% accuracy and robust performance against fabrication-induced corner rounding.^52^ Xu et al. utilized GAs to design aperiodic elastic metasurfaces, addressing the design freedom limitations of periodic subunit cells by optimizing columnar resonator heights for arbitrary phase and amplitude modulation.^50^ Similarly, Luo et al. achieved broadband coherent perfect absorption (CPA) by designing tungsten-based metasurfaces optimized via GA, attaining over 90% accuracy across a wide bandwidth.^33^ Jiang et al. applied GAs to optimize solar-thermal metasurfaces, achieving 93% accuracy in a double-layer titanium nitride and silicon nitride structure.^48^ Cai et al. demonstrated the use of GAs for designing nonlocal metasurfaces, enhancing the focusing efficiency of ultrathin transmissive metal lenses by optimizing electromagnetic coupling in non-periodic metallic lattice structures.^51^ Sui et al. combined GAs with topology optimization to create ultra-broadband polarization conversion metasurfaces, achieving frequency conversion from 8.0 to 30.0 GHz.^49^ Modified GAs, such as the adaptive generative algorithm by Jafar-Zanjani et al., have further demonstrated the expanded applicability of GAs in metasurface design, exemplified by the dual-element plasmonic reflectarray.^53^

The main disadvantage of GAs is that they converge slowly in high-dimensional design scenarios. Because GAs follow heredity and random variation, they depend on chance, so the convergence speed is relatively slow. In addition, because it is a non-convex optimization process, local optimal solutions are easy to fall into, and introducing diversity and randomness increases the computational cost. Due to their limited algebra and size, the dependence on the initial species selection is substantial, which makes the search difficult and consumes considerable resources. GAs are restricted by fundamental constraints that limit their ability to precisely manage individual design parameters. However, when gradient information is uncertain or unreliable, GAs can be a powerful tool for optimizing large non-convex designs.

#### PSO for forward design

##### PSO

The main challenge for GAs is their slow convergence in high-dimensional design. In contrast, PSO converges faster and more accurately than the GA, demonstrating its superiority in numerical optimization problems in academia and industry.^54^^,^^55^ PSO is also an evolutionary approach that starts with random solutions and finds the global optimal solution by following the current best values. It performs global searches by simulating natural phenomena, such as the flocking behavior of birds, optimizing problems through the collaborative efforts of individuals within the swarm (Figures 3B and 3C). The algorithm represents candidate solutions as particles that continually update their positions and velocities to search for the optimal solution within the solution space.^56^^,^^57^ Each particle changes direction according to its best historical and global positions, gradually approaching the optimum.

##### Application

PSO is widely employed in optimizing metasurface structures due to its efficient search capabilities, producing significant advancements across diverse designs. PSO models the collaborative search behavior of a swarm, with each particle representing a potential solution that iteratively adjusts its position based on local and global information, facilitating rapid convergence to optimal solutions.

**Figure 5:**
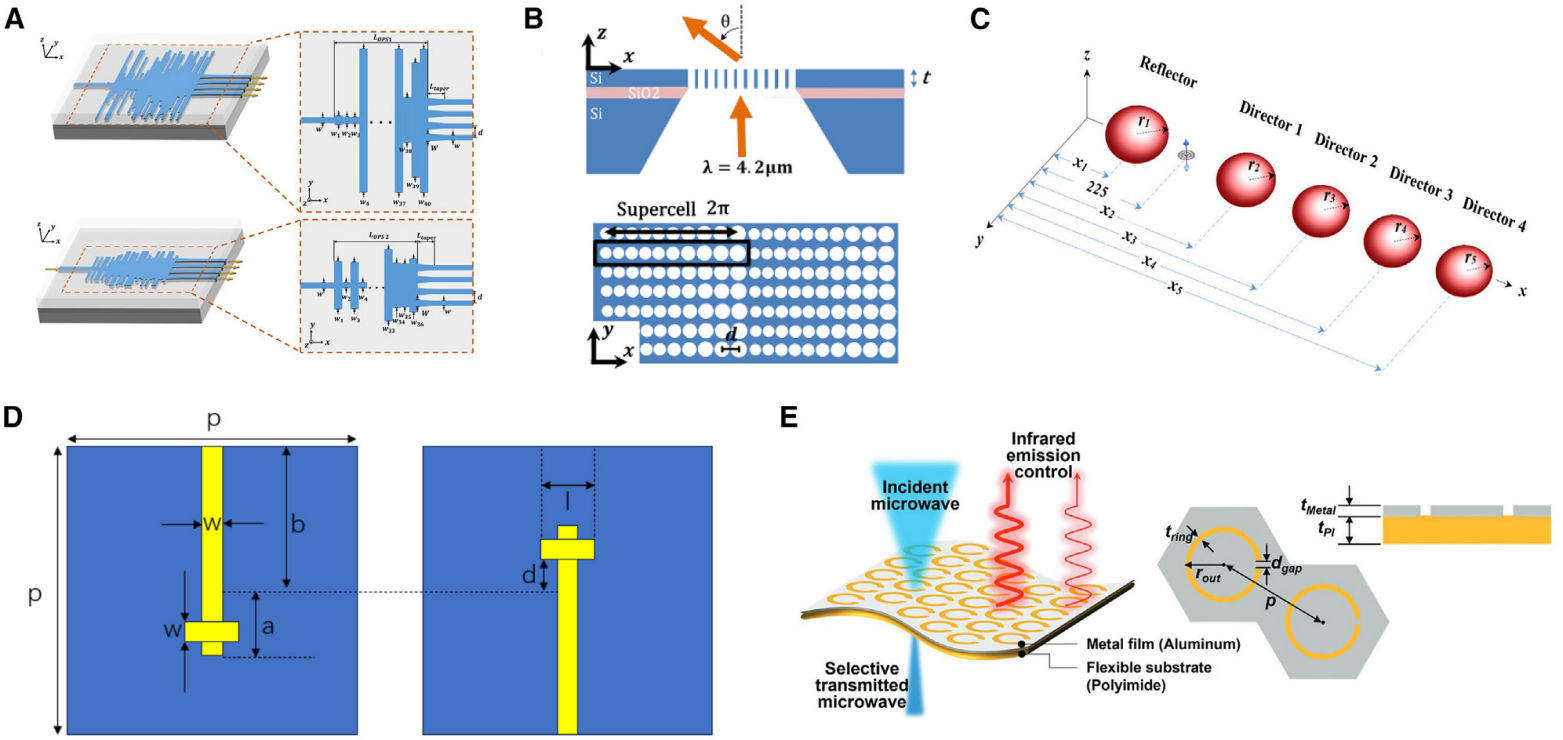
PSO adopted for the forward design of metasurface (A) Schematic of the designs OPS 1 and OPS 2. Reproduced with permission, from Kim et al. Copyright 2022,^59^ Paul Co Pub Consortium. (B) Schematic of a freestanding silicon nanohole array metasurface acting as a beam deflector with a deflect angle. Reproduced with permission, from Ong et al. Copyright 2017,^61^ Optical Society of America. (C) Conventional silicon optical Yagi–Uda nanoantenna. Reproduced with permission, from Mahmoud et al. Copyright 2017,^62^ Optical Society of America. (D) Configuration of the dual-layer element. Reproduced with permission, from Zhang et al. Copyright 2024,^63^ IEEE. (E) Schematic illustration of flexible compatible camouflage metasurface (FCCM). Reproduced with permission, from Nam et al. Copyright 2023,^64^ Wiley Online Library.

For example, as shown in Figure 5, Zhang et al. combined the equivalent circuit method (ECM) with PSO to design a toroidal metasurface, achieving a 30% improvement in broadband microwave absorption and visible light transparency.^31^ Jiang et al. introduced a physically assisted PSO (PA-PSO) algorithm for multi-feed lens antennas, achieving a 6-fold speedup compared to conventional PSO and effectively addressing multi-variable and multi-objective design challenges.^58^ Kim et al. employed PSO to develop compact 1 × 4 power splitters with low insertion loss and high uniformity, highlighting its computational efficiency and achieving acceptable accuracy.^59^ PSO has also demonstrated effectiveness in designing advanced metasurfaces with specific optical properties. Kildishev et al. utilized PSO to optimize negative refractive index metamaterials, benchmarking its performance against GAs and simulated annealing.^60^ Ong et al. designed a nanohole array metasurface optimized for beam deflection efficiency, achieving enhanced forward transmission and full 2 π phase control at mid-infrared wavelengths.^61^ This study identified scattering mechanisms associated with Kerker conditions to improve efficiency. Similarly, MAHMOUD et al. optimized nanoparticle-based Yagi antennas using PSO, enhancing directivity and achieving a 65.75% improvement in accuracy through ellipsoidal reflector designs.^62^

Further applications include adaptive optimization of metasurface units by Zhang et al., who used PSO combined with HFSS modeling to refine phase and amplitude characteristics, automating fitness calculations and parameter updates.^63^ Nam et al. leveraged PSO for flexible metasurfaces, achieving maximum infrared tunability and microwave selective transmission.^64^ Yu et al. fine-tuned nanofin rotation angles in gradient phase metasurfaces to improve polarization conversion efficiency, resulting in an improvement in design accuracy from 73.4% to 87.3%.^65^

In summary, the PSO algorithm has strong global search capabilities. However, in complex metasurface design, the information exchange between particles may lead to group convergence and cause it to become trapped in a local optimum. This algorithm has fewer parameters than the GA, but their values impact performance significantly. Incorrect parameter settings may cause the algorithm to converge more slowly and with lower accuracy. They may also fall into a local optimum trap. Further, PSO performance depends heavily on the distribution of the initial population, so an unreasonable distribution of the initial population may prevent a successful search. However, some strategies help prevent a local optimum trap. These include exchanging information between global and individual optima to jump out of the local optimum and search for a better solution. By improving the weight factor, larger weights lead to better global convergence, while smaller weights have stronger local convergence. As the number of iterations increases, the inertial weight gradually decreases, and by linearly decreasing the weight, the problem of falling into a local optimum can be avoided. Also, improving the learning factor can utilize the contraction factor to control the system behavior and lead to convergence.

#### ACO for forward design

##### ACO

The ACO algorithm is a heuristic optimization method inspired by the foraging behavior of ants and has recently been applied to metasurface design. ACO explores and optimizes the design space by simulating how ants mark paths with pheromones when searching for food.^32^ In metasurface design, each potential design is treated as a path, while the pheromone concentration represents the quality of the design. The algorithm increases the pheromone concentration along superior design paths through multiple iterations, guiding more ants to follow these paths and optimizing the design parameters globally. This approach is well-suited for multi-objective optimization problems in metasurface design, such as balancing electromagnetic performance metrics like transmission, reflection, and phase control (Figure 3D). The strength of ACO lies in its powerful global search capability, which effectively avoids becoming trapped in a local optimum and leads to superior design solutions.^66^ Further, the parallel nature of ACO makes it highly effective in large-scale design problems, enabling it to find optimal solutions within a reasonable time. In summary, ACO is a robust optimization tool for metasurface design, particularly for complex tasks that require efficient search and global optimization.

##### Application

The ACO has been extensively applied to optimize the structural arrangement, geometry, and material properties of metasurfaces, enhancing their electromagnetic, optical, and acoustic responses. The algorithm excels in navigating complex design spaces, managing high-dimensional, nonlinear, and multi-objective optimization challenges, reducing design cycles, and improving metasurface functionality.^67^^,^^68^ Despite limitations such as slow convergence, sensitivity to parameters, and susceptibility to local optima, ACO’s adaptability, global search capabilities, and inherent parallelism underscore its value in metasurface optimization.

**Figure 6:**
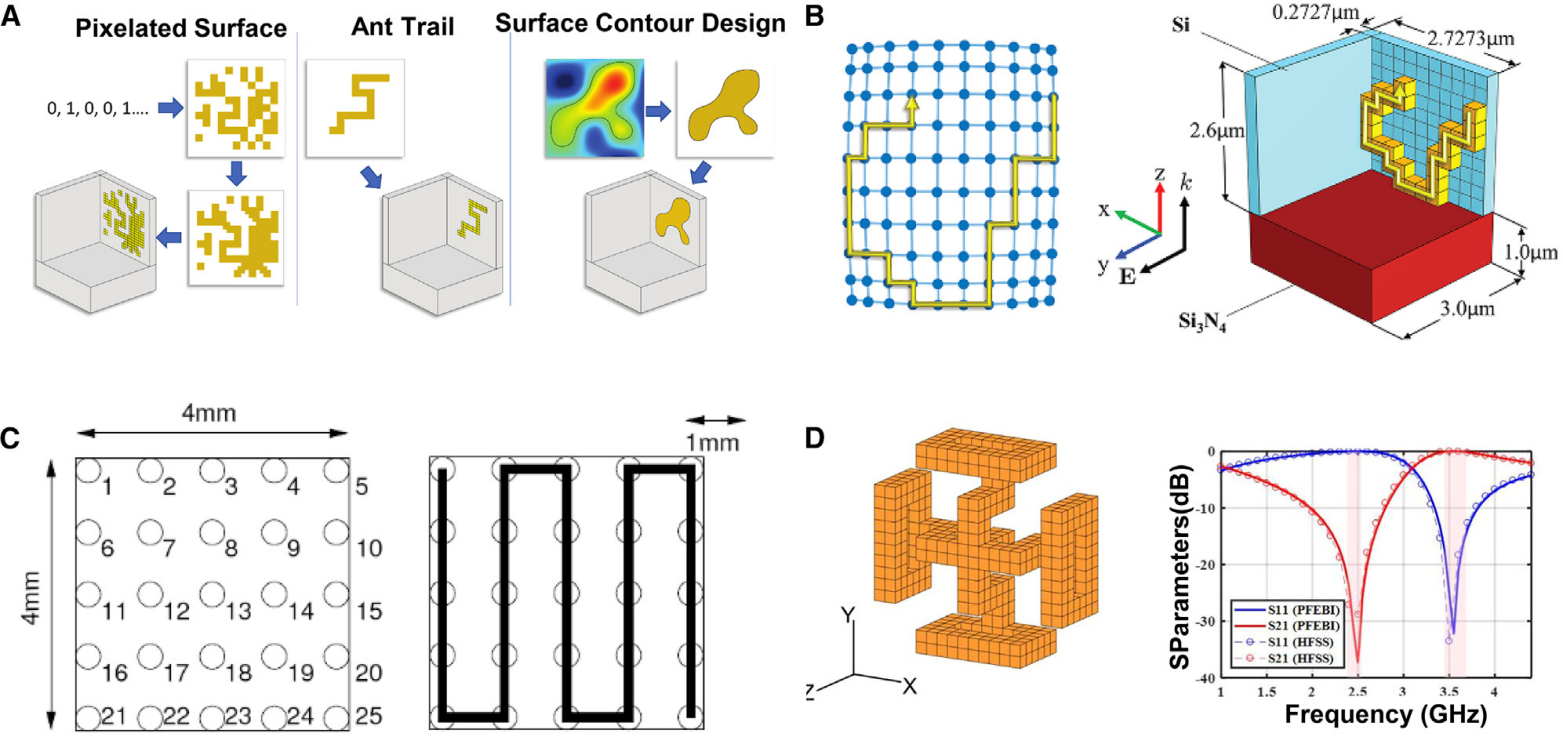
ACO adopted for the forward design of metasurface (A) Shows how a gold trace could be formed by starting at some point and then allowing an ant to wander in different directions and terminate at a random length. Reproduced with permission, from Whiting et al. Copyright 2020,^70^ METAMORPHOSE VI AISBL. (B) Generalized graph representation of 7 × 7 × 1 and 7 × 7 × 2 grids mapped to different physical geometries. Reproduced with permission, from Zhu et al. Copyright 2019,^34^ American Chemical Society. (C) Defines the grid and numbering system and shows a possible meander line antenna. Reproduced with permission, from Lewis et al. Copyright 2009,^71^ Optical Society of America. (D) 3-D loaded Jerusalem cross generated by MOLACO. Reproduced with permission, from Zhu et al. Copyright 2017,^72^ IEEE.

As shown in Figure 6, Zhang et al. employed an improved binary ant colony optimization (IBACO) algorithm, integrating competitive and instructional learning mechanisms, for the design of a dual-frequency terahertz (THz) metamaterial absorber sensor.^69^ This approach addressed the limitations of traditional methods that rely heavily on prior knowledge, achieving accuracy levels exceeding 97%, along with enhanced sensitivity. Whiting et al. utilized the lazy ant colony optimization (LACO) algorithm to develop metasurface elements for three-dimensional membrane projection lithography (MPL).^70^ Through this method, they designed a gold-based beam-steering device in the mid-infrared range, achieving wider fields of view, broader bandwidths, and higher transmission efficiency. Additionally, the LACO algorithm effectively tackled challenges associated with large design spaces and complex coupling effects.

Zhu et al. expanded the capabilities of ACO with the multi-objective lazy ant colony optimization (MOLACO) algorithm to facilitate the inverse design of 3D plasmonic metasurfaces for mid-wave infrared (MWIR) applications.^34^ This approach revealed non-intuitive metal-dielectric structures with superior phase response and transmission performance, comparable to all-dielectric planar designs. Furthermore, MOLACO was utilized to optimize 3D frequency selective surfaces (FSS), enhancing solution diversity through adaptive colony shielding and lazy ant mechanisms.^72^ Lewis et al. demonstrated the efficacy of Ant Colony System (ACS) algorithms in optimizing compact serpentine RFID antennas, balancing efficiency and resonant frequency while managing the complexities of multi-objective antenna optimization.^71^

The ACO algorithm’s distributed computing and decentralized control features significantly affect continuous metasurface design. ACO is unsuitable for discrete structural unit design. The algorithm randomly selects the next node, so it may take excessive time to produce positive feedback, and the algorithm converges slowly. The ACO algorithm employs positive feedback, by which initial solutions are initially built randomly, and the quality of the solutions influences the evolution of the optimal solution. A poor initial solution can cause the algorithm to become stuck in a local optimum. The algorithm has many interrelated parameters, so parameter selection is based on experience or trial and error. Poorly chosen initial parameters weaken the algorithm’s optimization and slow its convergence. The ACO algorithm has a tradeoff between population diversity and convergence speed. The better the population diversity, the higher the probability of obtaining the global optimal solution, but the longer the search time. Some strategies, such as algorithm structure improvement and selecting an appropriate population size to avoid slow convergence, improve the ACO algorithm. Optimizing algorithm parameters and setting a reasonable taboo list during path planning can reduce the algorithm’s efficiency, as can improving the information update rule and updating the pheromone heuristic factor based on the search results.

#### DBS for forward design

##### DBS

The DBS algorithm is an optimization method used in the design of optical components, known for its scalability and simplicity, making it widely favored by metasurface researchers. The core idea of the DBS algorithm is to optimize the structure to achieve specific optical performance goals iteratively.^73^^,^^74^ It is particularly suited for discrete binary design problems, where the design parameters (such as the presence or absence of material in a metasurface) are binary, typically represented as “0” and “1”. The basic process starts with a random or predefined binary initial structure, where each pixel has a value of either “0” or “1”. The optical performance of this structure is then evaluated using methods such as finite-difference time-domain (FDTD) or rigorous coupled-wave analysis (RCWA). Next, each pixel is checked sequentially, temporarily flipping its value and reassessing the optical performance of the modified structure. If the performance improves, the change is retained; otherwise, the pixel value is reverted to its original state. By adjusting the design parameters pixel by pixel and evaluating their impact on the target performance, the algorithm gradually converges on an optimized structure configuration. This process ultimately yields an optimized metasurface design, well-suited for binary design tasks, where discrete changes in the structure significantly influence the overall performance (Figure 3A).

##### Application

**Figure 7:**
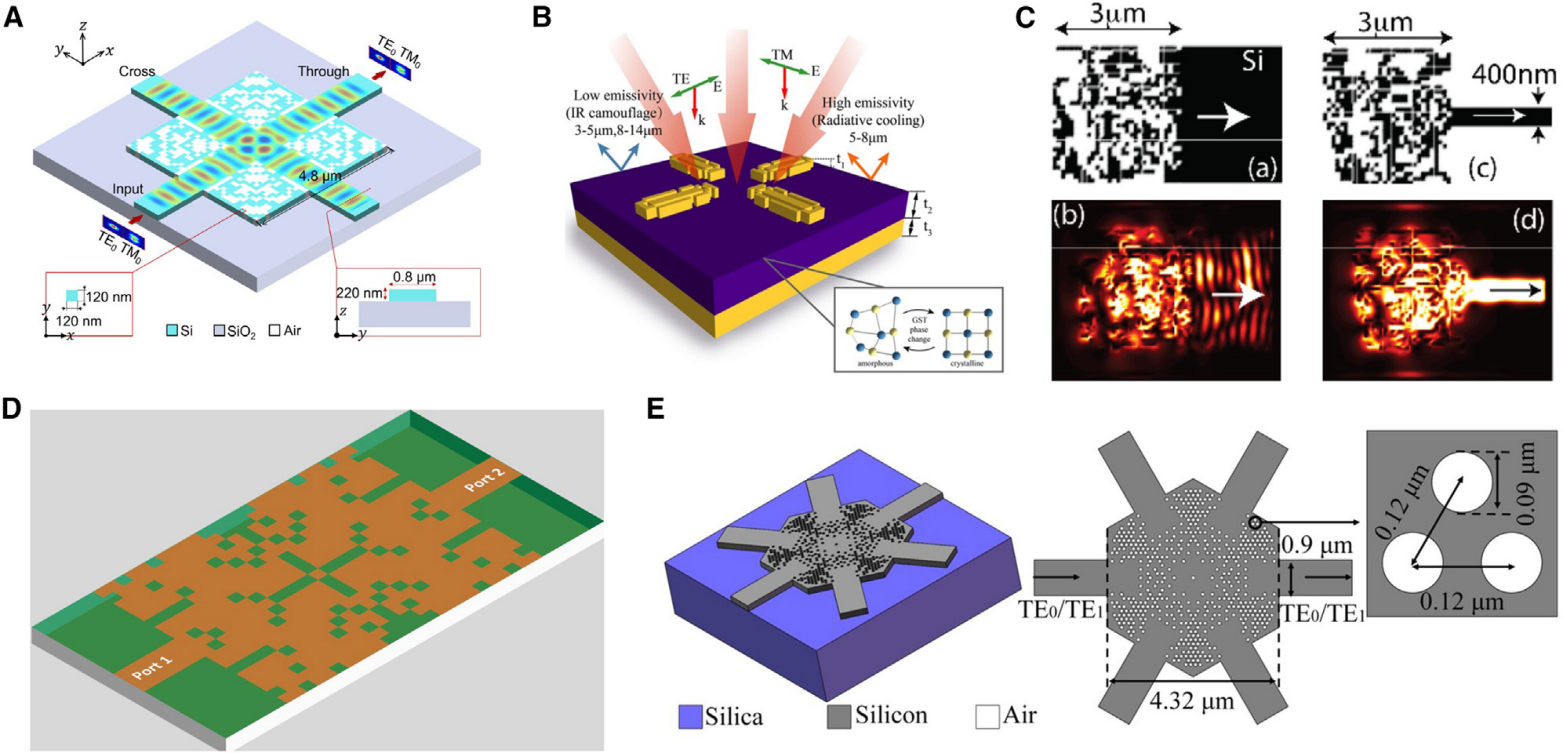
DBS adopted for the forward design of metasurface (A) The 3D schematic diagram of the proposed polarization-insensitive waveguide crossing based on dielectric metasurface. Reproduced with permission, from Liu et al. Copyright 2024,^75^ IEEE. (B) Schematic of the PCM-based MSE. The MSE is developed with inverse design gold grating coated on the GST and gold mirror. Reproduced with permission, from Jiang et al. Copyright 2022,^77^ Optical Society of America. (C) Free-space to multi-mode waveguide coupler and corresponding simulated time-averaged intensity distribution. Reproduced with permission, from Shen et al. Copyright 2014,^78^ Optical Society of America. (D) Example of a pixelated RF planar surface. Reproduced with permission, from Lee et al. Copyright 2023,^79^ IEEE. (E) The three-channel and dual-mode waveguide crossing. Reproduced with permission, from Ma et al. Copyright 2023,^76^ Optical Society of America.

The DBS algorithm is a powerful tool in metasurface design, enabling the optimization of structural arrangements and material distributions to achieve specific optical functionalities. As shown in Figure 7, Liu et al. employed the DBS algorithm to design a dielectric metasurface-based ultra-compact, polarization-insensitive silicon waveguide crossing device.^75^ The resulting design exhibited low insertion loss and crosstalk across a broad wavelength range with an ultra-small footprint of 4.8 × 4.8 μm^2^, demonstrating excellent accuracy. Similarly, Ma et al. used the DBS algorithm to develop ultra-compact multi-channel, multi-mode waveguide crossing devices, overcoming limitations of conventional designs in achieving compactness and multi-channel, multi-mode performance simultaneously.^76^ Their work included three-channel and four-channel dual-mode waveguide crossings, advancing the field of waveguide integration.

Lee et al. applied DBS to optimize pixelated surfaces for dual-band RF filters, achieving significant reductions in design area while enhancing frequency response.^79^ This efficiency and accuracy make DBS a valuable approach for realizing complex RF functionalities, such as dual-band filtering. Jiang et al. utilized the algorithm to design nanostructured metallic patterns for a tunable mid-infrared selective emitter, tailored for applications in infrared stealth and thermal management.^77^ Meanwhile, Shen et al. optimized three distinct metamaterial couplers using DBS, achieving higher coupling efficiency, broader bandwidths, and substantial size reductions—shrinking device footprints by an order of magnitude compared to previous best-performing designs.^78^

The strength of the DBS algorithm lies in its simplicity and ease of implementation, rendering it well-suited for optimizing complex structures with numerous design degrees of freedom. However, its computational efficiency is relatively low, particularly in large-scale design problems, where point-by-point searches lead to lengthy computation times. To improve efficiency, DBS can be combined with other optimization algorithms, such as GAs or Simulated Annealing, to expedite convergence. In conclusion, the DBS algorithm remains a powerful tool for optimizing metasurface structures with binary design parameters, facilitating high-precision optical component design.

### Gradient algorithm for forward design

#### AM for forward design

##### AM

The adjoint method (AM) is a mathematical technique employed to solve complex optimization problems, particularly those constrained by partial differential equations (PDEs).^80^ The core concept of the Adjoint Method is to simplify the computation of the gradient of the objective function, especially when the parameter space is large. In optimization problems, the objective function typically depends on design parameters and is constrained by system states, typically described by PDEs.^81^^,^^82^ By solving an adjoint equation, the method reduces gradient calculation complexity from being proportional to the number of design parameters to being proportional to the number of state variables, significantly improving computational efficiency.

##### Application

Originally developed for control problems,^83^ the AM has become widely applied in aerospace and mechanical engineering,^84^ particularly in shape and topology optimization. More recently, AM has been adopted for metasurface design, significantly enhancing design efficiency. For effective gradient-based metasurface design, two key factors must be considered: first, a powerful and accurate electromagnetic solver is essential, as gradient-based methods typically require multiple gradient computations, which can be computationally expensive; second, the objective function must be continuously differentiable.^85^^,^^86^ By leveraging a single forward and adjoint simulation, the shape derivative across all spatial points can be calculated, thereby improving the efficiency of the objective function evaluation.^87^^,^^88^

**Figure 8:**
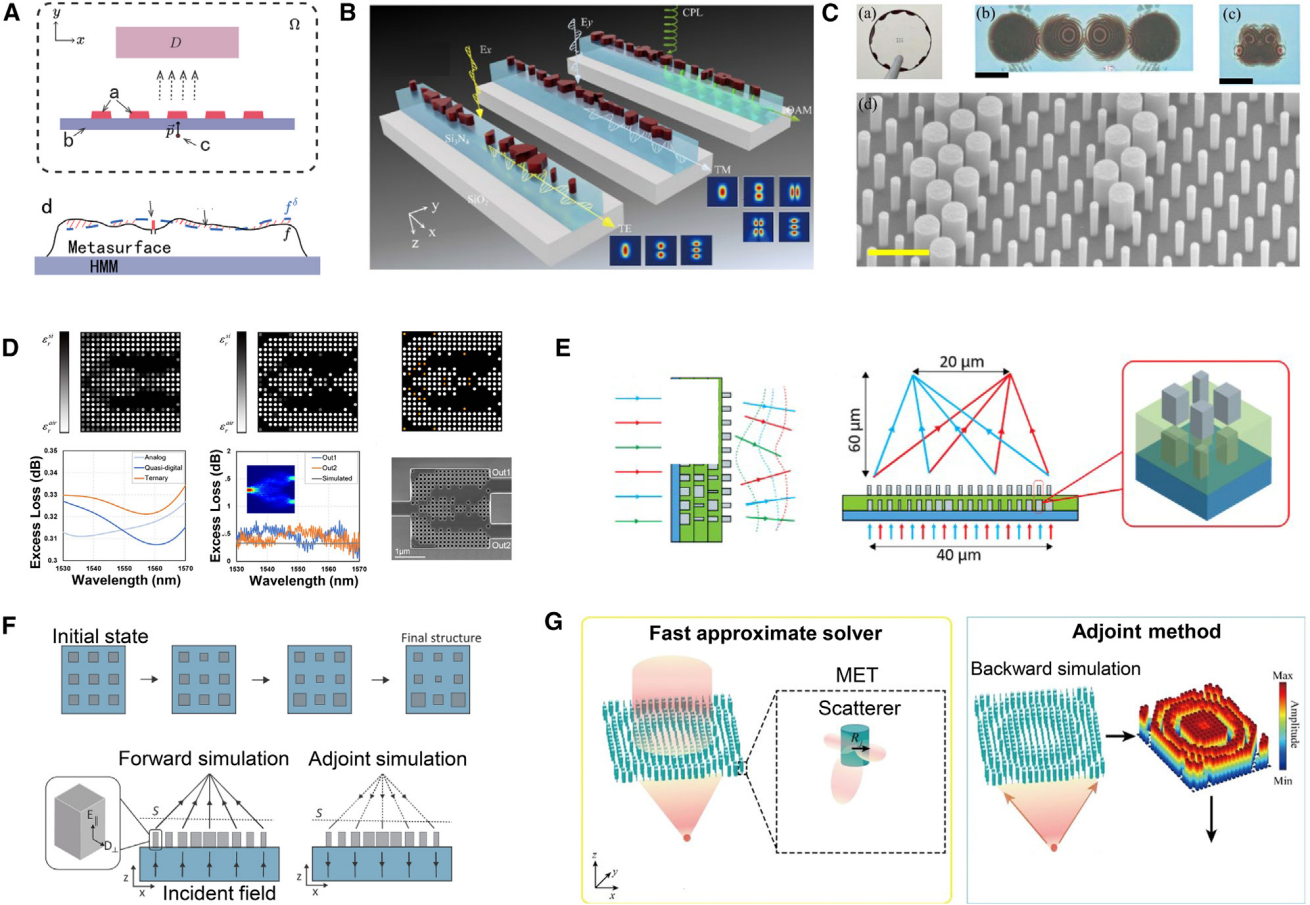
AM adopted for the forward design of metasurface (A) Setup for the spontaneous emission model. Reproduced with permission, from Ma et al. Copyright 2024,^90^ The Optical Society. (B) PRandSOCinfreeform metasurface-assisted unidirectional waveguide coupler. Reproduced with permission, from Chen et al. Copyright 2024,^91^ American Chemical Society. (C) Fabricated metasurface couplers. Reproduced with permission, from Oh et al. Copyright 2024,^89^ Optical Society of America. (D) 3 dB power divider. The optimized analog and quasi-digital patterns in the first and second stages. Reproduced with permission, from Wang et al. Copyright 2020,^85^ OSA & CLP. (E) Schematic of a metastructure with the ability to generate independent wavefronts for different wavelengths. Reproduced with permission, from Mansouree et al. Copyright 2020,^92^ Optical Society of America. (F) Parameterized optimization: meta-atom dimensions are updated but constrained to a simple shape. Reproduced with permission, from Mansouree et al. Copyright 2021,^36^ American Chemical Society. (G) Schematic of the MET-adjoint method. Reproduced with permission, from Zhang et al. Copyright 2022,^93^ American Chemical Society.

As shown in Figure 8, Ren et al. introduced a digitized AM approach to design a single-mode low-noise power splitter and a dual-mode demultiplexer.^85^ Through a three-stage optimization process, they successfully designed high-performance components. Similarly, Oh et al. applied AM to design a metasurface-based free-space coupler, optimizing the coupling of single-mode fibers (SMFs) with multi-core fibers (MCFs).^89^ By optimizing the phase distribution of the metasurface, they maximized the overlap between the output light field and the MCF core modes, demonstrating the potential of AM in photonic applications.

Ma et al. used the AM algorithm to design a grating metasurface combined with hyperbolic metamaterials (HMMs), significantly improving the efficiency of spontaneous emission (SE).^90^ By formulating the design problem as a PDE-constrained optimization and solving it efficiently using gradient descent and the adjoint state method, they achieved exceptional results. Liu et al. extended the application of AM to realize full-space electromagnetic wave manipulation using a single-layer metasurface, allowing independent control of transmitted and reflected light fields via local interference effects of individual nanostructures.^35^ Chen et al. employed adjoint-based topology optimization to design a freeform metasurface capable of unidirectionally coupling free-space light into customized waveguide modes with polarization control and mode selection.^91^ Zhang et al. further enhanced the versatility of AM by integrating multipole decomposition with adjoint optimization, presenting a novel approach for optimizing multifunctional metasurfaces with tailored electromagnetic functionalities.^93^ These studies illustrate the power of adjoint optimization as an efficient numerical tool for optimizing complex metasurface structures.

Building on these developments, Zhou et al. introduced a design framework based on coupled-mode theory to model wave dynamics in metasurfaces with high accuracy.^94^ By implementing adjoint optimization for large-scale metasurface design, they significantly reduced computational complexity while maintaining precise predictions of the metasurface’s far-field distribution. Mansouree et al. proposed a parametric metasurface design method using adjoint optimization, where sub-wavelength meta-atoms are employed to transform incident light.^36^ This approach reduced computational costs and enabled the design of large-scale, high-performance metasurfaces. Mansouree et al. extended adjoint optimization to 2.5D metasurface structures, where stacked layers allow for independent control of multiple wavelengths on the same surface, thereby enhancing the functionality and performance of optical components.^92^

Heuristic algorithms can be quite resource-intensive and time-consuming when dealing with metasurfaces that have complex geometries or functions, resulting in high computational costs and unacceptable delays. In such cases, gradient-based adjoint algorithms are often more advantageous. For metasurfaces with continuous parameters, adjoint methods can efficiently meet performance targets. The key difference between these approaches lies in how they update the objective function. Heuristic algorithms typically employ random search methods to explore the design space and identify improved solutions, while adjoint algorithms use gradient-based update strategies to optimize the objective function. This gradient-based approach can be more efficient for continuous parameter optimization, as it systematically adjusts design parameters based on calculated gradients to enhance performance.

#### LST for forward design

##### LST

Level set optimization (LST) algorithm is a specialized optimization method for metasurface design. It focuses on locally modifying the topology of metasurface unit structures to enhance the overall electromagnetic performance.^95^^,^^96^ The core idea of LST is to iteratively adjust the geometry or material distribution within localized regions of the metasurface to optimize its electromagnetic response while preserving the characteristics of the local structure.^97^ This approach enables precise and efficient optimization by targeting specific areas of the design.^98^^,^^99^

##### Application

The LST algorithm excels in scenarios requiring precise electromagnetic wave manipulation, mainly when dealing with complex functionalities and multi-parameter adjustments. By using implicit functions to parameterize the design regions, LST can accurately track topological changes at the boundaries. Additionally, it can easily constrain the minimum feature size, which improves robustness and ensures manufacturability.

**Figure 9:**
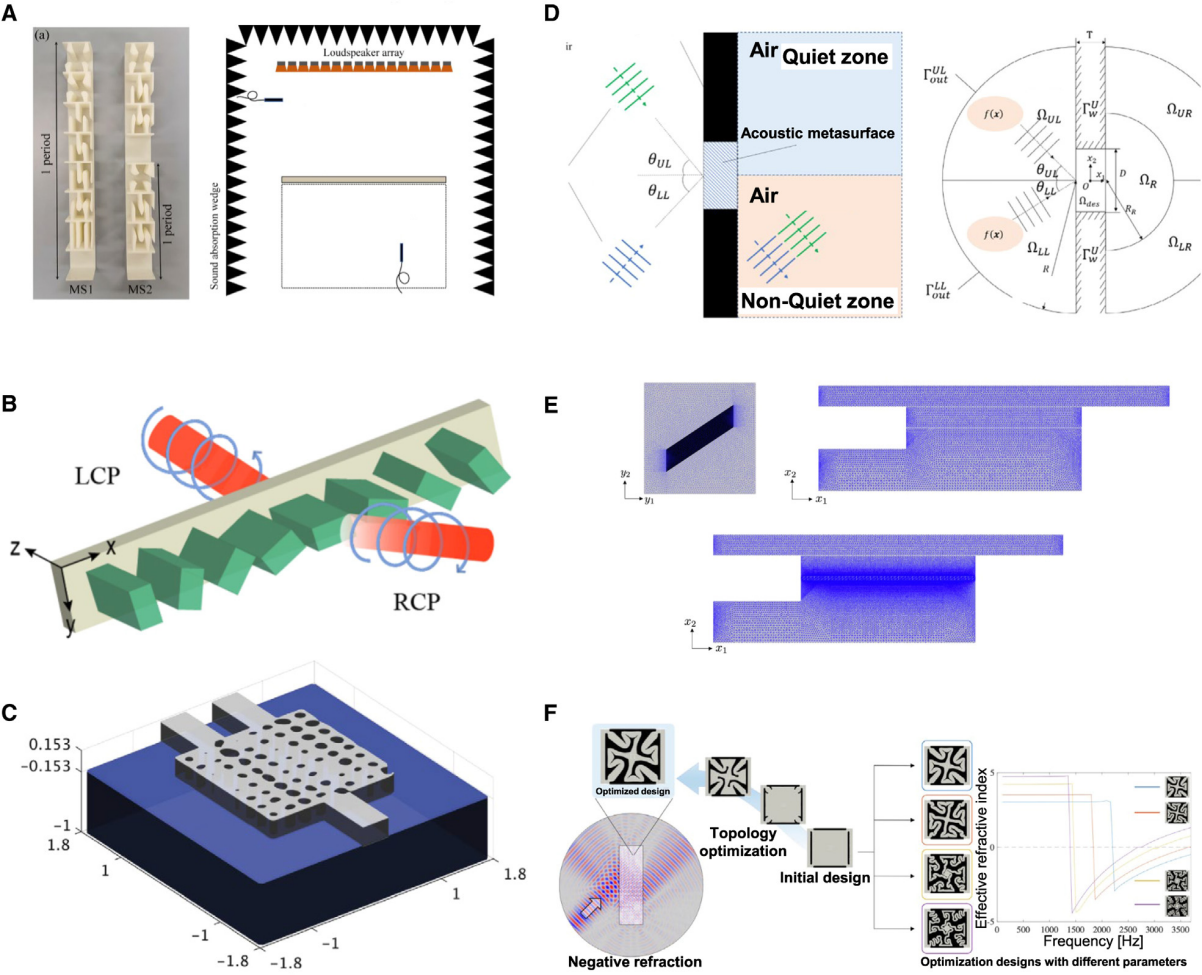
LST adopted for the forward design of metasurface (A) The MS1 and MS2 samples fabricated by 3D printing technique. Reproduced with permission, from Guo et al. Copyright 2024,^100^ The Optical Society. (B) Initial discrete geometric phase metasurface for high-efficiency beam deflection based on multi-objectives level-set optimization. Reproduced with permission, from Dong et al. Copyright 2022,^104^ The Optical Society. (C) Optimized design and details of the optimization process of the shape of a duplexer. Reproduced with permission, from Lebbe et al. Copyright 2019,^98^ Elsevier. (D) Schematic illustration of the function of the metasurface Geometrical settings. Reproduced with permission, from Emoto et al. Copyright 2023,^101^ Elsevier. (E) Finite element discretization for the microscale analysis, macroscale analysis with homogenization, and reference analysis. Reproduced with permission, from Noguchi et al. Copyright 2021,^105^ Elsevier. (F) History of the objective functions and immediate designs. Reproduced with permission, from Noguchi et al. Copyright 2022,^102^ Elsevier.

As shown in Figure 9, In 2024, Guo et al. used the level-set topology optimization method to develop an acoustic metasurface aimed at precise control of transmitted wavefronts.^100^ By designing periodically arranged unit cells, they created a linear phase gradient in the transmitted waves, enabling efficient sound wave modulation. Emoto et al. applied level-set topology optimization to design an acoustic metasurface for regional sound isolation.^101^ By optimizing shape and material distribution, they achieved an acoustic metasurface structure capable of blocking sound waves incident from any angle across a broad frequency range from propagating to specific areas. In 2022, Noguchi et al. used level-set topology optimization to design a maze-like acoustic metamaterial structure that realizes broadband double negativity in the low-frequency range, significantly improving computational efficiency.^102^ In 2022, Guo et al. also employed level-set topology optimization to design an acoustic metasurface structure for reflection wavefront modulation, allowing for arbitrary control of reflected waves.^103^ Dong et al. utilized multi-objective level-set topology optimization to design a quasi-continuous acoustic surface for efficient beam steering.^104^ They optimized the structure of an initially discrete geometric phase acoustic surface to enhance the beam steering efficiency. In 2021, Noguchi et al. also used level-set optimization for acoustic metasurface design, optimizing unit structures to achieve the desired macroscopic acoustic performance.^105^ Lebbe et al. applied the level-set method for shape and topology optimization, designing robust nanophotonic devices and proposing a multi-objective optimization algorithm addressing wavelength and geometric shape uncertainties.^98^

The LST algorithm’s advantages include reduced design complexity by iteratively modifying only part of the structure, significantly lowering computational costs, and enabling fine-tuning of critical areas to improve precision and performance. It is flexible and suitable for multifunctional and multi-parameter designs, offering progressive optimization that avoids the computational bottlenecks of global optimization. However, LST can encounter issues with local optima, which may prevent achieving global optimal solutions, and its effectiveness is dependent on the initial structure and region selection. The complexity of implementation also necessitates strong computational resources and specialized knowledge.

#### DTO for forward design

##### DTO

The DTO algorithm is also a gradient algorithm widely used for metasurface design. In DTO, the design region is typically discretized into pixels, where each pixel can be filled with either solid dielectric material or air. In DTO, the material permittivity of each pixel is represented as a continuous value to facilitate gradient computation. The process includes initial design domain setup, material density variable definition, objective function formulation, physical model solving, sensitivity analysis, density updating, and iterative optimization. This method allows for gradual optimization of the metasurface’s topology, achieving precise control of electromagnetic waves and finding applications in optical device design, stealth technology, and wavefront control.

##### Application

**Figure 10:**
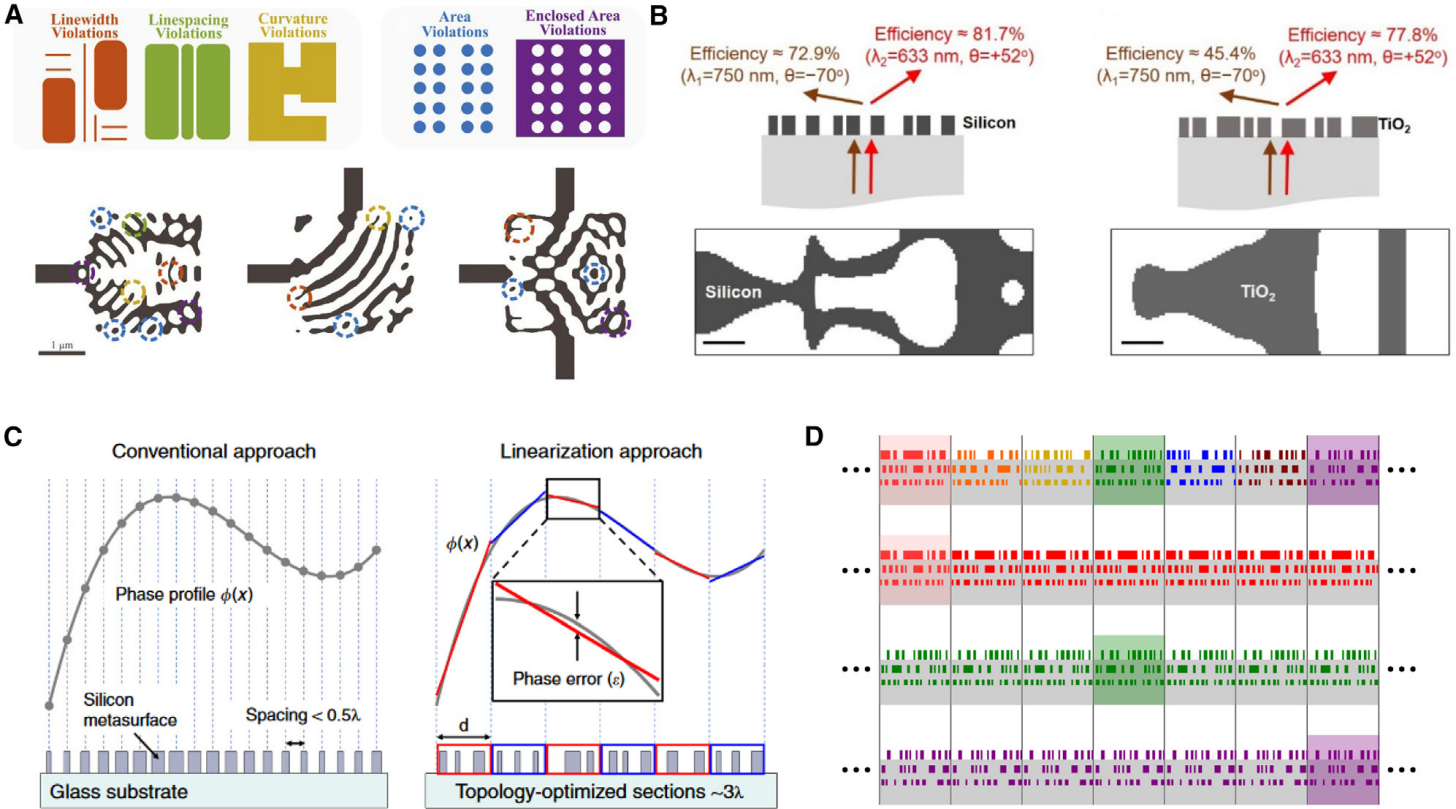
DTO adopted for the forward design of metasurface (A) Five fundamental design rule constraints for semiconductor foundries. Reproduced with permission, from Hammond et al. Copyright 2021,^106^ Optical Society of America. (B) Comparison of metagrating wavelength-splitters based on crystalline-silicon at visible wavelengths. Reproduced with permission, from Yang et al. Copyright 2017,^108^ Optical Society of America. (C) Conventional approaches sample the desired phase profile at discrete points and specify phase-shifting elements to form a nanoscale phased array. Reproduced with permission, from Phan et al. Copyright 2019,^109^ Springer Nature. (D) An arbitrary aperiodic multi-layered meta-structure (top) is approximated by solving a set of periodic scattering problems (bottom). Reproduced with permission, from Lin et al. Copyright 2019,^107^ Optical Society of America.

As shown in Figure 10, Hammond et al. proposed an automated design method based on DTO, integrating design constraints such as minimum linewidth and line spacing.^106^ They also demonstrated how to use differentiable morphological transformations to enhance the device’s robustness against over-etching and under-etching while satisfying manufacturing constraints. Lin et al. introduced a method combining topology optimization with local periodic approximation for designing large-area freeform optical metasurfaces.^107^ This approach optimizes optical elements with millions of degrees of freedom, overcoming the limitations of traditional subwavelength unit designs. Yang et al. employed topology optimization to design a single-layer transmissive grating and analyzed the relationship between material permittivity, device thickness, and beam deflection angle.^108^ They systematically studied the impact of different dielectric materials on the optical metasurface’s performance, especially in the visible and near-infrared frequency ranges. Phan et al. designed large-area, high-efficiency optical metasurfaces using topology optimization, significantly improving computational efficiency.^109^ By dividing the metasurface into smaller sections for individual optimization and then assembling them, they reduced computational complexity from a high polynomial order to linear.

DTO algorithms are highly efficient techniques for designing metasurface structures. They enable optimization across thousands or millions of degrees of freedom, each representing a “pixel” of the manufactured device, allowing for the discovery of designs with unprecedented performance. However, the vast design freedom of topology optimization is constrained by practical manufacturing processes, and various approaches have been employed in previous studies to constrain the minimum length scales and curvatures in the optimized designs.

#### Fusion method for forward design

Forward design methods for optical metasurface structures usually involve heuristic optimization algorithms, such as GA, PSO, and gradient optimization.^110^ This is mainly due to the rapid acquisition of optical properties of non-patterned structures through simulation.^111^^,^^112^^,^^113^ However, metasurface optical simulation usually takes a significant amount of time, so relying solely on them for metasurface inverse design is very limited, especially considering multiband constraints.^114^ Recently, deep learning (DL)^115^ has been widely used in the inverse design of hypersurfaces. The fusion method provides a new way to solve complex design challenges by combining the nonlinear mapping capability of DL with the global search capability of optimization technology. Neural networks can be used to quickly predict the physical response of the cell surface while optimization algorithms globally optimize design parameters. A neural network model is first trained to quickly evaluate optical performance at different structural parameters. A GA uses the predicted result as a fitness function and finds the optimal structure that satisfies the design goal through iterative search. This method dramatically reduces the time required for numerical simulation while retaining the global search capability of GAs.

##### Application

**Figure 11:**
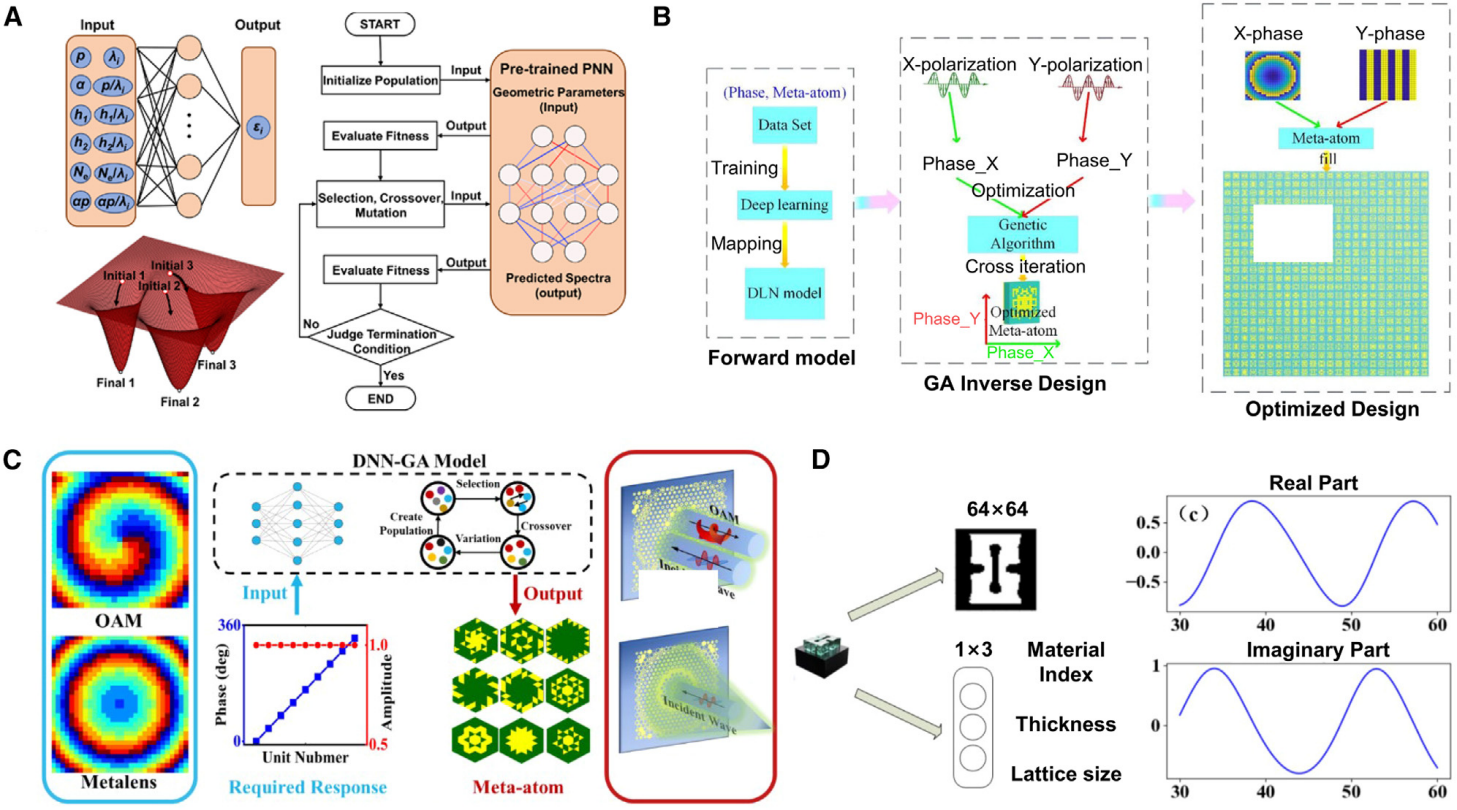
Fusion algorithm adopted for the forward design of metasurface (A) The framework of PNN-GA. Reproduced with permission, from Wang et al. Copyright 2024,^116^ Elsevier. (B) The process of this paper: firstly, establishing the DLN as a forward model; then using the GA to optimize the meta-atom via a forward model; finally, using the optimized meta-atom to design the meta-atom. Reproduced with permission, from Zhu et al. Copyright 2020,^118^ IOP Publishing Ltd. (C) The flow chart of the DNN-GA optimization. Reproduced with permission, from Wu et al. Copyright 2022,^119^ Optical Society of America. (D) The meta-atom is expressed by 2D structural images and structural parameters. Reproduced with permission, from Yu et al. Copyright 2022,^120^ Optical Society of America.

As shown in Figure 11, Wang et al. combined Physics-Driven Neural Networks (PNN) with GAs to design an infrared camouflage metasurface for laser applications.^116^ In this PNN-GA framework, PNN serves as the solver, while GA is employed for design optimization, resulting in an AZO-Ge disk metasurface that achieves invisibility at a 1.06 μm laser wavelength. The metasurface also provides mid-infrared thermal camouflage and effective thermal management. Christopher Yeung et al. proposed a framework integrating adjoint optimization with machine learning to overcome local optima issues in non-convex design spaces.^117^ This framework first employs automated machine learning (AutoML) for global search approximation, followed by adjoint optimization for local refinement, significantly enhancing design performance.

Naseri et al. combined machine learning with PSO to design inhomogeneous bianisotropic metasurfaces, demonstrating a hybrid approach to optimizing complex metasurface structures. Zhu et al. integrated DL with GAs to design multifunctional metasurface apertures.^118^ They used deep learning networks (DLNs) as forward models and GA to optimize the metasurface’s phase response, enabling multifunctional control of electromagnetic waves. Yu et al. introduced a method combining machine learning with GAs for high-dimensional metasurface design.^120^ By utilizing PNN to evaluate the fitness of individuals in GA quickly, this approach avoids local optima and enables efficient exploration of the design space, achieving global solutions.

Wu et al. combined DNN with GAs for rapid metasurface function design.^119^ They used DNN to classify and optimize the initial GA population, restricting initial phases to within 30°, which significantly improved optimization efficiency and reduced the number of iterations required.

Thus, combining machine learning with optimization algorithms improves the efficiency of the forward design of metasurfaces and resolves some of the limitations of traditional methods. These include problems in high-dimensional complex design, cost calculation, design flexibility, and design efficiency. By combining machine learning models, such as DNNs and VAEs, with GAs and PSO, the powerful prediction ability of DL can significantly reduce the complexity of high-dimensional parameter space search. Optimization algorithms dramatically reduce computational resource requirements that rely directly on physical simulations by searching based on DL predictions. Combining neural networks and optimization can better deal with complex problems, such as multi-objective optimization, multi-function requirements, and dynamic design. By reducing the number of simulations and optimization iterations, a hybrid model provides an efficient solution for large-scale metasurface design.

## Inverse design of metasurface

DL has profoundly impacted autonomous driving,^121^ robotic control,^122^ language translation,^123^ audio recognition,^124^ and image classification.^125^ It was introduced in metasurface design as a powerful method for studying complex mappings of microstructures and response characteristics. DL is a data-driven approach that uses DNNs to extract features from datasets without human intervention.^126^ It can establish complex mappings from inputs (structural parameters) to outputs (optical properties) through training on labeled datasets, thus rapidly and accurately determining the optical properties of metasurfaces through these mappings.^127^^,^^128^ Unlike the forward design methods described earlier, which require extensive iterative electromagnetic (EM) simulations and time, DL is highly efficient and can almost instantaneously provide a well-trained neural network for designing metasurface structures.^129^^,^^130^ There are two main approaches in DL to accelerate metasurface research models. The first is to use neural networks as surrogate solvers to predict optical responses in a brief time. The second is to have neural networks output the required design parameters directly, based on the target response. This section introduces various DL model architectures applied to metasurface structural design and reviews classical works that use DL techniques to address inverse design problems.

In analyzing the performance index of inverse design algorithms, the inverse is usually evaluated in terms of parameter freedom, applicable data type, accuracy, and efficiency. Table S2 compares the performance indicators of different inverse design algorithms. These indexes can clarify the advantages and disadvantages of various algorithms in hypersurface design so that reasonable selection can be made based on common factors such as calculation cost and accuracy. Table S4 shows the computing requirements of various neural network algorithms, including those of different algorithms on data complexity and computing resources. The inverse design algorithm uses AI-based DL models, such as CNNs, DNNs, VAEs, RNNSs, and GANs. These algorithms rely on massive training data and efficient model training methods to provide high-precision design prediction with limited computational resources. When DL models, such as CNNs and DNNs, are trained on large-scale datasets, they can extract complex patterns and features and perform well in large-scale metasurface design. However, training DL models requires much computation, especially when datasets are large. GPUs and distributed computing environments become critical. Generative models such as VAEs and RNNSs can manage nonlinear mapping and timing data more efficiently through coding-decoding structures or recursion mechanisms. However, they also require powerful computational resources to train and process the model. Especially in large-scale metasurface designs, these inverse design algorithms may need to be fine-tuned to the model’s parameters to ensure optimal computational efficiency and maintain accuracy.

### Convolutional neural network for inverse design

#### CNN

CNN is a type of DL model widely used for image recognition, classification, and feature extraction. Their main characteristic is their ability to effectively extract spatial features and local patterns from data through a combination of convolutional layers, pooling layers, and fully connected layers.^131^^,^^132^^,^^133^ CNN has significant applications in metasurface structural design. By encoding the geometric parameters or electromagnetic response characteristics of metasurface structures as input data, CNN can automatically learn and extract key feature patterns in the design, allowing them to predict the performance of metasurface structures or reverse-engineer design parameters that meet specific requirements.^134^ This application greatly enhances the efficiency of metasurface design, especially in complex design spaces, where CNN can quickly identify effective design solutions, reducing the time spent on trial-and-error methods traditionally used.

#### Application

**Figure 12:**
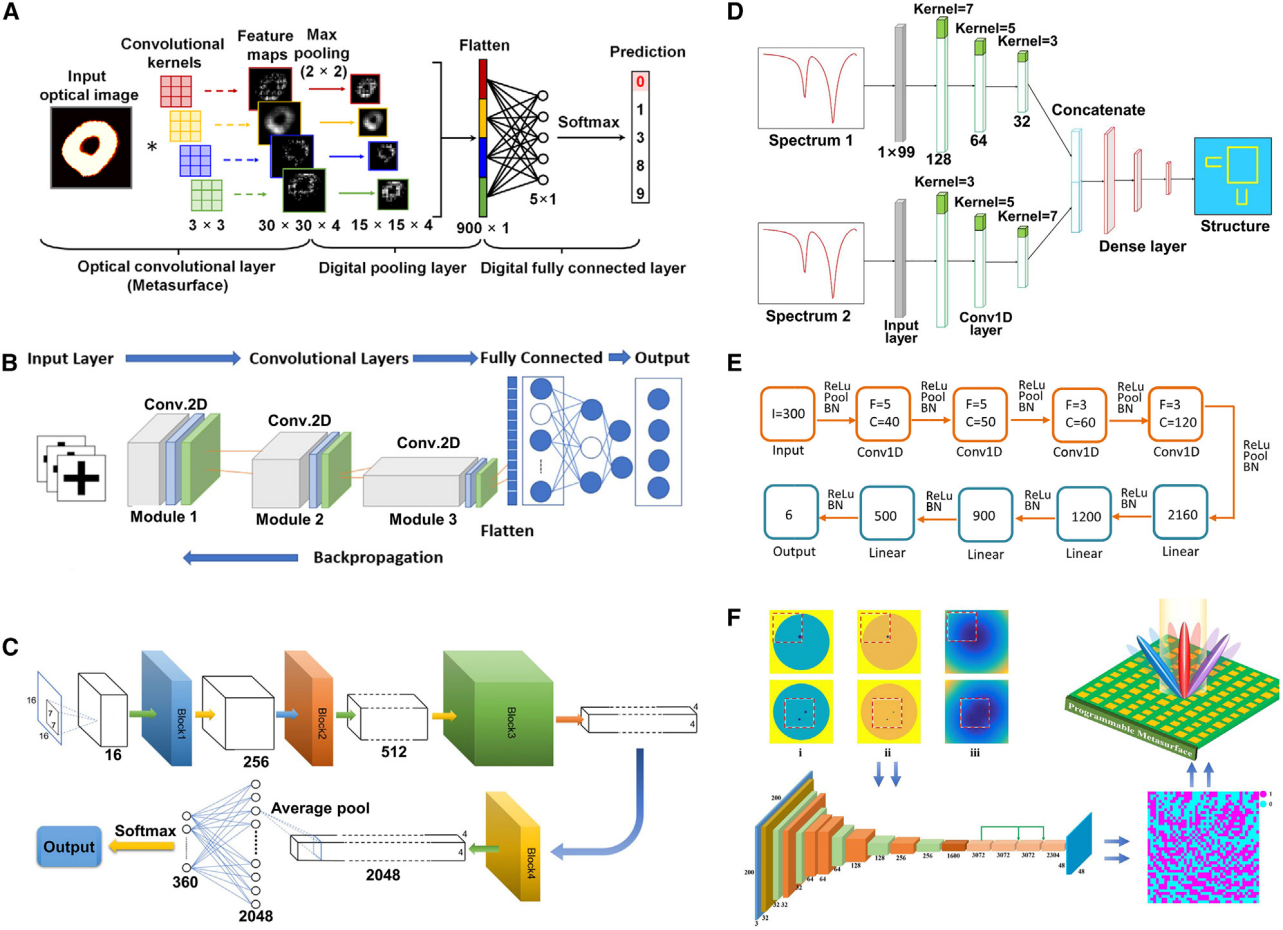
CNN adopted for the inverse design of metasurface (A) The architecture of the metasurface-based optical CNN. Reproduced with permission, from Luo et al. Copyright 2024,^136^ Wiley Online Library. (B) Schematic of CNN architecture. Reproduced with permission, from Razi et al. Copyright 2023,^138^ Elsevier. (C) The graphical representation of the CNN structure. Reproduced with permission, from Zhang et al. Copyright 2019,^135^ Wiley Online Library. (D) Illustration of inverse neural network architecture. Reproduced with permission, from Zhu et al. Copyright 2022,^139^ IOP Publishing Ltd. (E) Detailed parameters of the CNN. Reproduced with permission, from Lin et al. Copyright 2020,^141^ Optical Society of America. (F) Schematic of a coding scheme for programmable metasurface based on deep learning techniques. Reproduced with permission, from Noguchi et al. Copyright 2020,^140^ IEEE.

As shown in Figure 12, Zhang et al. trained a CNN to predict the reflection phase of double anisotropic meta-atoms at specific frequencies when illuminated by transverse electric (TE) and transverse magnetic (TM) polarized waves.^135^ The binary encoding of digital meta-atoms (0 and 1) provides a natural parameterization method that can be directly used as CNN input. Luo et al. utilized CNNs to design an optical accelerator for metasurfaces, achieving sub-picosecond delay and nearly zero energy consumption.^136^ The proposed optical accelerator has a very small volume of only 0.016 cubic millimeters, with extremely low crosstalk (−20 dB) and polarization insensitivity, capable of performing multiple convolution operations in parallel and extracting various features from optical encoded images. Zhang et al. used deep CNNs to achieve forward prediction of three complex meta-surface patterns in the microwave range (including arbitrary polygonal connections, basic mode combinations, and fully random binary patterns) for simulating reflectance.^137^ Razi et al. employed a CNN-based optimization method to design and optimize terahertz broadband metasurface patterns to enhance the efficiency of tandem solar cells.^138^ By training on over ten thousand different geometric shapes of metasurfaces using the CNN model, they selected the best metasurface structures to improve light capture and electromagnetic response. Zhu et al. proposed a CNN-based optimization method for the design of electromagnetically induced transparency (EIT) metasurfaces.^139^ The cascade CNN structure used demonstrated excellent performance in extracting spectral features, achieving efficient inverse design while maintaining a low mean squared error (MSE). Shan et al. applied deep CNNs to the encoding computation of metasurface units, enabling spatial and temporal modulation of complex electromagnetic waves.^140^ Their DL method was able to compute the encoding matrix of metasurface units within milliseconds, with prediction accuracy exceeding 94%. Lin et al. introduced a CNN-based inverse design method for plasmonic metasurfaces.^141^ By using the target spectrum as input, CNNs could quickly and accurately predict the key parameters that determine the geometry of the metasurface, achieving higher accuracy and generalization capability, especially in handling complex design problems with multiple parameters. It also effectively addressed data inconsistency issues arising from symmetry.

Applying CNNs to metasurface design offers significant advantages, including automatic extraction of complex features, efficient prediction of design performance, and the ability to handle large-scale datasets. This allows effective solutions to be rapidly identified in complex metasurface design spaces and significantly improves design efficiency. However, CNNs also have drawbacks, such as a dependence on large amounts of labeled data, long training times, complex network structure design, and potential overfitting in certain design tasks, which may limit their performance. Future developments may focus on reducing dependence on labeled data and further enhancing the adaptability and flexibility of CNN in design applications.

### Deep neural network for inverse design

#### DNN

DNN is a neural network architecture with multiple hidden layers that can capture and learn complex nonlinear relationships, making them widely applicable in various data-driven prediction and classification tasks.^142^ In metasurface structural design, DNN can effectively predict the performance of metasurface structures or reverse-engineer design solutions that meet specific requirements by processing the design parameters or electromagnetic response data through multiple layers of nonlinear transformations and feature extraction.^47^^,^^143^^,^^144^ This approach significantly simplifies the complex numerical simulations and optimization processes traditionally used in the design, enhancing efficiency and accuracy. Additionally, DNN allows for the directional design of bidirectional structures, improving the efficiency of both forward and inverse problem-solving. When adequately trained, these models exhibit high accuracy and efficiency.

#### Application

DNNs utilize training datasets composed of feature vectors and corresponding labels to model the relationship between device structures and optical responses. One approach leverages forward networks to predict the optical responses of integrated photonic devices with high accuracy, effectively serving as a computationally efficient approximation to numerical simulations in iterative optimization processes. Alternatively, inverse models are employed, where the optical response is provided as input, and the corresponding device structure is generated as output. Several techniques, such as cascaded networks, dimensionality reduction methods, and deep convolutional mixed-density networks, have been proposed to tackle the many-to-many relationship challenges in inverse models.

**Figure 13:**
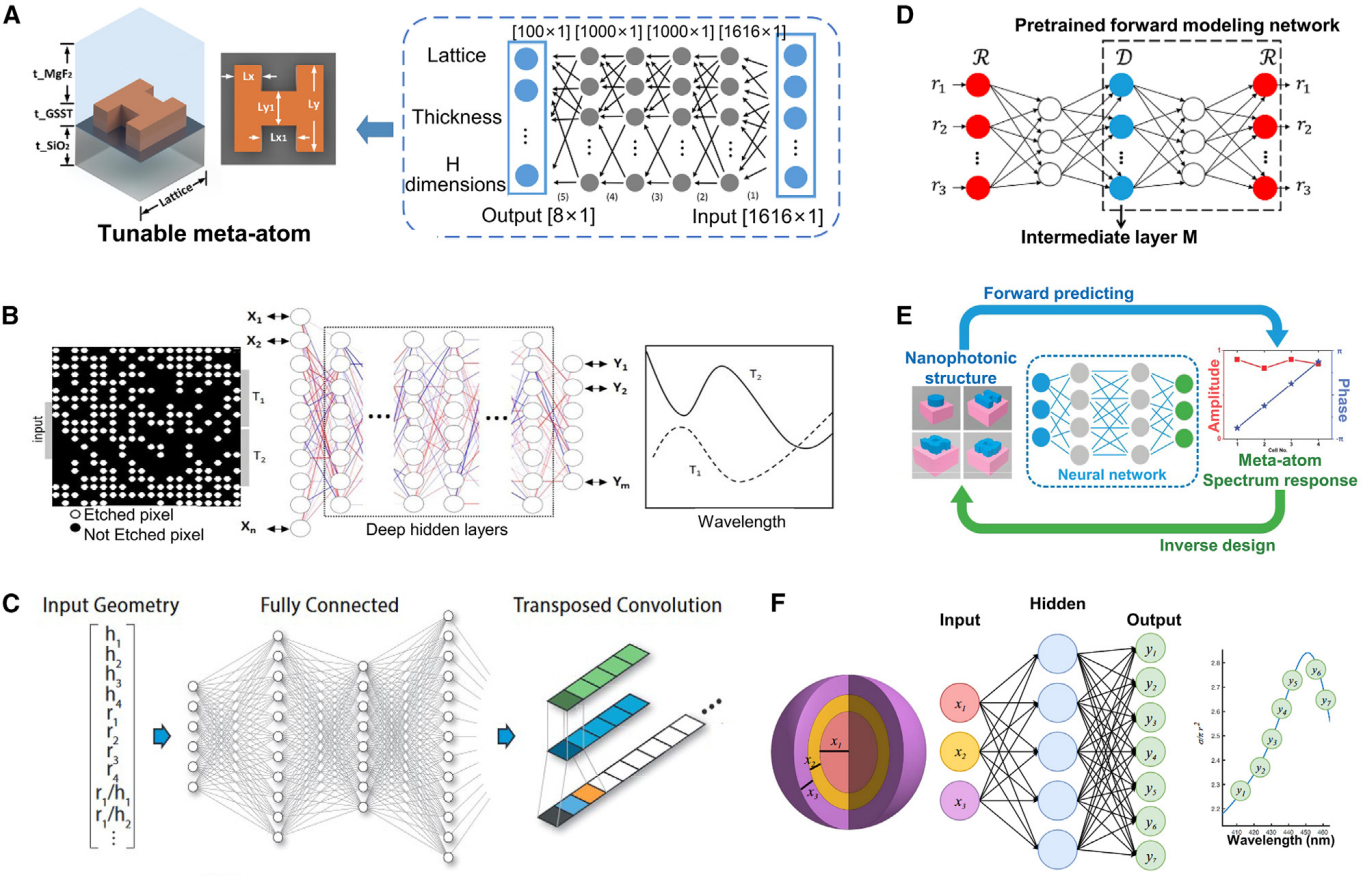
DNN adopted for the inverse design of metasurface (A) The meta-atom inverse DNN constructed based on fully-connected neural networks. Reproduced with permission, from An et al. Copyright 2022,^42^ De Gruyter. (B) The DNN can take device topology design as input and spectral response of the metadevice as label or vice versa. Reproduced with permission, from Kojima et al. Copyright 2019,^147^ Springer Nature. (C) Fully connected layers are fed a set of geometric inputs. Reproduced with permission, from NADELL et al. Copyright 2019,^148^ Optical Society of America. (D) The tandem network is composed of an inverse design network connected to a forward modeling network. Reproduced with permission, from Liu et al. Copyright 2018,^149^ American Chemical Society. (E) Flowchart of the closed-loop meta-atom design network. Reproduced with permission, from An et al. Copyright 2019,^150^ American Chemical Society. (F) The NN architecture has as its inputs the thickness of each shell of the nanoparticle, and as its output the scattering cross section at different wavelengths of the scattering spectrum. Reproduced with permission, from Peurifoy et al. Copyright 2018,^38^ AAAS.

As shown in Figure 13, Wang et al. proposed a generative elimination framework based on reversible DNNs.^145^ By introducing additional trainable potential variables during the inverse training process, they minimized the impact of phase-free far-field patterns, effectively avoiding one-to-many mapping issues. Both simulation and experimental results demonstrated a 50% improvement in accuracy over traditional DNNs, while maintaining a competitive process cycle. Additionally, Wang et al. introduced a fully automatic inverse-forward design method for multi-electromagnetic (EM) structural designs, utilizing a demand-based multi-inverse forward neural network (M-IFNN).^146^ This method, employing parallel combination techniques, achieved 100% correct prediction of the corresponding structure and significantly reduced the mean absolute error (MAE) in inverse predictions.

An et al. developed a DNN-based design method for embedded active metasurface photonic devices.^151^ By combining DNN with scattering matrix (S-matrix) optimization, they simplified computational burdens while achieving precise, target-driven designs. Similarly, Tahersima et al. utilized DNN for the inverse design of integrated photonic power splitters, training the network to design splitters with over 90% transmission efficiency on a micrometer-scale silicon-on-insulator (SOI) platform.^147^

Nadell et al. proposed a DNN-based accelerated design method for all-dielectric metasurfaces.^148^ By inputting geometric parameters and related physical knowledge, the network efficiently predicts scattering parameters, significantly accelerating design processes. They also introduced a fast forward dictionary search (FFDS) method to address the one-to-many mapping problem in inverse design. In another study, An et al. proposed a target-driven DNN-based design method for all-dielectric metasurfaces, addressing the computational efficiency and accuracy issues of traditional methods for designing complex metasurfaces.^150^ This approach simultaneously predicts amplitude and phase responses, enabling the design of various metasurface functionalities such as filters, phase modulators, and reconfigurable metasurfaces.

Liu et al. introduced a cascaded training method for inverse design based on DNNs.^149^ By connecting a pre-trained forward network to the inverse design network’s output, forward prediction errors serve as supervisory signals, guiding the inverse retrieval output. This indirect training method effectively addresses data inconsistency issues arising from the non-uniqueness of inverse design problems. Peurifoy et al. used DNNs to simulate light scattering by multilayer nanospheres and solved the structural inverse design problem of metasurfaces through backpropagation.^38^ The network, consisting of four fully connected layers with 250 neurons each, was trained with spectra from 50,000 scatterers with randomly varied nanocapsule thicknesses. The trained network successfully generated accurate profiles from scattering spectra of randomly shaped geometries.

In summary, a DNN exhibits powerful nonlinear modeling capabilities in metasurface design, capturing complex relationships between design parameters and performance and significantly improving design efficiency and accuracy, especially in high-dimensional and complex multivariable design problems. However, DNNs also have drawbacks, including the need for large amounts of high-quality training data, high computational costs during training, susceptibility to overfitting, and poor model interpretability. These limitations may restrict their application in metasurface design.

### Recurrent neural networks for inverse design

#### RNN

RNN is primarily used for analyzing natural language and time-series data.^152^ These networks analyze sequences of data to detect internal links within the data. The network’s output is fed back into the input layer, allowing it to remember past conditions and create new data sequences.^153^ This characteristic makes RNN suitable for modeling wave phenomena, such as acoustic-optic effects, as these physical phenomena can be viewed as a simulation of RNN. Such properties make RNN particularly effective for time-domain simulations of optical signals or spectra and for metasurface design.

#### Application

RNN has broad potential applications in metasurface structural design. Various RNN-based models, such as long short-term memory (LSTM) networks and transformers, have been developed to handle time-series data efficiently and accurately. Additionally, the gated recurrent unit (GRU),^154^ a gating mechanism within RNNs driven by LSTM units,^155^ is simple, computationally robust, and advantageous in addressing common gradient vanishing issues associated with RNN.

**Figure 14:**
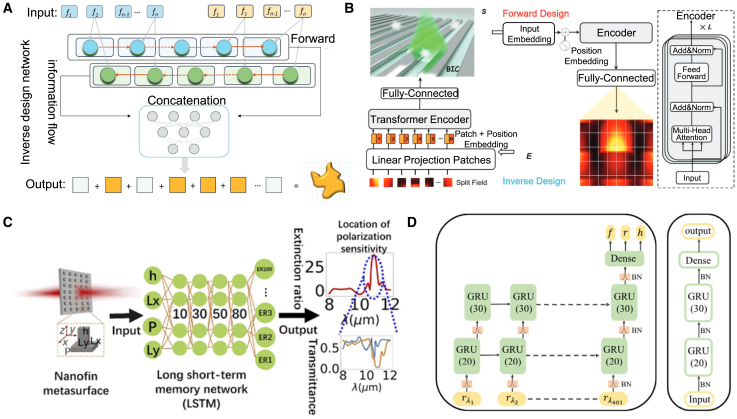
RNN adopted for the inverse design of metasurface (A) Revealing spectral connection for metasurface inverse design. Reproduced with permission, from Zhang et al. Copyright 2024,^156^ Wiley Online Library. (B) Schematic drawing for the architecture of transformer-based model. Reproduced with permission, from NADELL et al. Copyright 2024,^158^ Wiley Online Library. (C) LSTM as predicting network. Reproduced with permission, from Deng et al. Copyright 2022,^160^ Springer Nature. (D) Architecture of the GRUs inverse design model. Reproduced with permission, from Yan et al. Copyright 2021,^159^ IOP Publishing Ltd.

For instance, as shown in Figure 14, Zhang et al. proposed an inverse design method based on a bidirectional gated recurrent unit (Bi-GRU) network for optimizing metasurface design.^156^ By comparing unidirectional RNNs, attention-based transformer structures, and traditional fully connected neural networks, they demonstrated the effectiveness of the bidirectional mechanism. Mao et al. utilized an RNN-based transformer model for the inverse design of ultra-broadband terahertz polarization converters.^157^ This model achieved a fast and precise inverse design, producing a polarization converter corresponding to the target spectrum in just 0.006 s. Chen et al. introduced a transformer-based DL approach for the design of all-dielectric surface-enhanced Raman scattering (SERS) metasurfaces, leveraging the strong coupling characteristics of quasi-bound states (Q-BIC) to enhance SERS effects.^158^ The Q-BIC-driven all-dielectric SERS metasurface not only achieved higher local field enhancement but also significantly expanded the enhancement region, surpassing metal-based SERS. Yan et al. employed deep RNN-based methods for inverse design and spectral prediction of metasurfaces.^159^ By extracting sequence features of spectra, they accurately predicted and designed metasurface structure parameters, particularly excelling in handling long sequence data. Deng et al. proposed a method using LSTM neural networks for the direct inverse design of nanofin metasurfaces.^160^ By applying LSTM for prediction and inverse design, they rapidly and accurately predicted the polarization sensitivity of nanofin metasurfaces and directly derived the geometric parameters of nanofins based on target extinction ratio (ER) values.

In summary, the powerful time-sequence processing capability of RNNs can assist in analyzing and predicting the optical and electromagnetic performance of metasurface elements. By training with input data related to material parameters, geometric structures, and functional requirements, RNNs can generate optimized design solutions and enhance metasurface performance. Compared to traditional design methods, RNNs can effectively reduce design cycles and find optimal solutions among complex design parameters, making them suitable for multifunctional and tunable metasurface design fields.

### Variational autoencoder for inverse design

#### VAE

VAE is a type of DL model widely used in metasurface design for generating and optimizing complex structural designs. VAE employs an encoder-decoder architecture to map high-dimensional design data into a continuous latent space where samples can be generated and optimized.^161^ In metasurface design, VAE can learn latent structural features from large datasets of existing designs, enabling the generation of new metasurface structures. By adjusting variables in the latent space, designers can explore different design options and optimize the electromagnetic performance of metasurfaces.

#### Application

VAE provides powerful generative and optimization capabilities for metasurface design. By encoding the design parameters of a metasurface into the latent space, VAE can effectively capture complex distribution features within the design and generate diverse structural design options. This approach allows for the generation of new metasurface structures through the decoder and facilitates interpolation in the latent space for performance optimization and multi-objective design.

**Figure 15:**
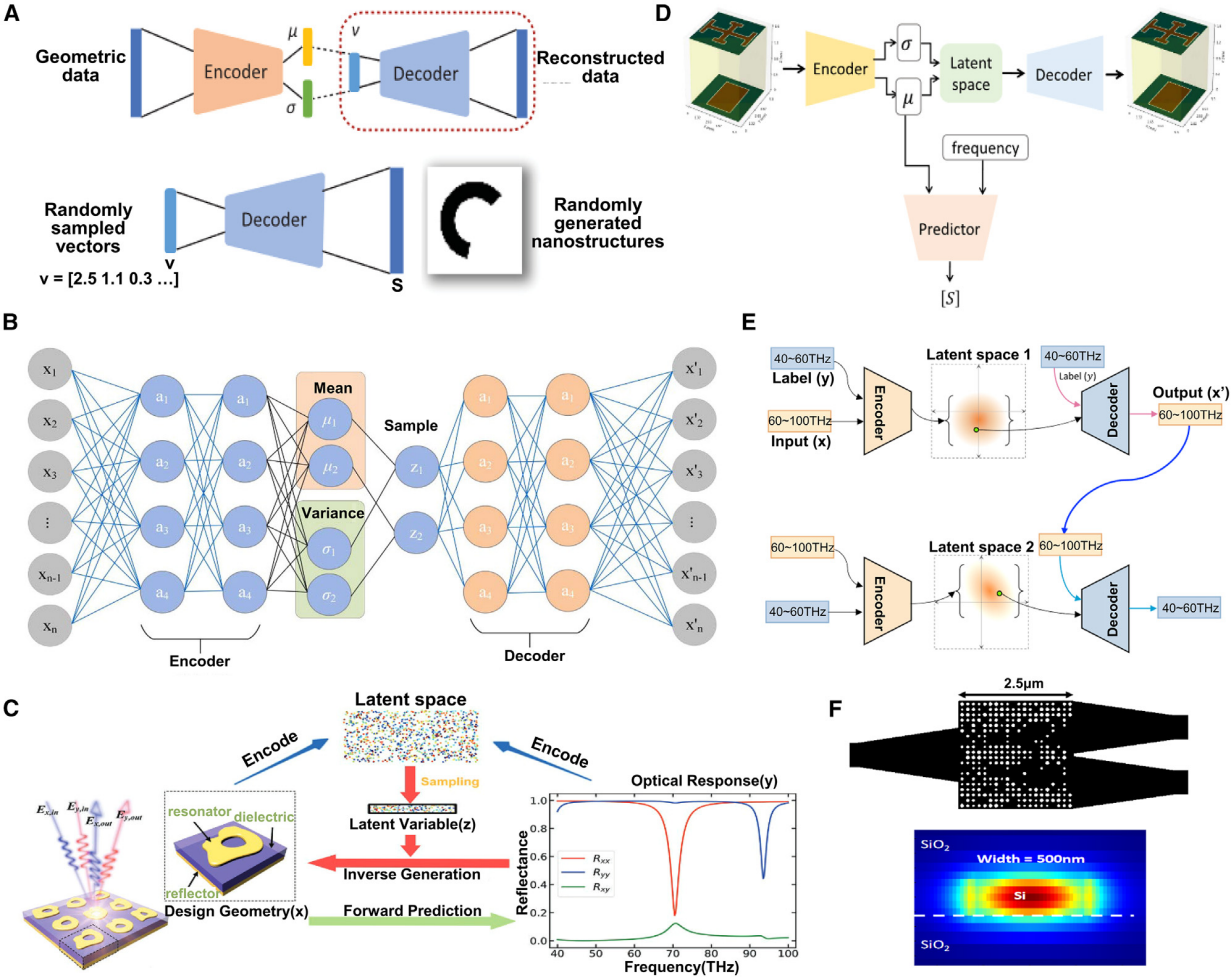
VAE adopted for the inverse design of metasurface (A) Basic architecture of a VAE implemented in the framework. Reproduced with permission, from Liu et al. Copyright 2020,^166^ IEEE. (B) Entire variational autoencoder architecture. Reproduced with permission, from Wei et al. Copyright 2012,^163^ IEEE. (C) The proposed deep generative model for metamaterial design and characterization. Reproduced with permission, from Ma et al. Copyright 2019,^37^ Wiley Online Library. (D) Proposed approach to regularize a latent space where both the physical shape of the metasurfaces and their scattering properties change smoothly for better optimization. Reproduced with permission, from Naseri et al. Copyright 2021,^164^ IEEE. (E) A macro perspective to look at the architecture of the proposed network consisting of two cascaded networks, namely, generation network and elimination network, each of which is composed of an encoder, the latent space, and a decoder. Reproduced with permission, from Chen et al. Copyright 2023,^162^ Nature Publishing Group. (F) Power splitter footprint & cross-section of the input/output waveguide. Reproduced with permission, from Tang et al. Copyright 2020,^165^ Optical Society of America.

As shown in Figure 15, Chen et al. proposed a generative-elimination framework based on VAE to derive complex metasurface spectral designs, addressing the limitations of traditional forward and inverse design methods in many-to-many mappings.^162^ They used VAE to generate multiple candidate spectra and selected the best candidates through an elimination network, enabling inference from low to high-frequency spectra. Wei et al. combined VAE with equivalent circuit theory to accelerate metasurface generative design.^163^ By compressing high-dimensional data into a lower-dimensional latent space and adaptively selecting more targeted training samples, the optimization process became more efficient. Naseri et al. employed VAE methods for designing multi-layer metasurfaces, representing and optimizing the physical structures and scattering characteristics of metasurfaces in a low-dimensional continuous latent space to simplify and accelerate the design process.^164^ This approach combined machine learning predictive models with simulation models to automatically generate metasurface structures that meet specific scattering properties. Tang et al. used a conditional variational autoencoder (CVAE) and adversarial review model to design a multi-stage nano-optical broadband power splitter.^165^ By incorporating adversarial blocks to optimize CVAE, the generated power splitter achieved high broadband performance. Liu et al. proposed a hybrid strategy combining VAE with improved evolutionary algorithms for rapid discovery and design of metasurface structures.^166^ VAE encoded all possible unit structures into a continuous latent space, and evolutionary strategies were used to optimize and identify the optimal vectors meeting design goals. Ma et al. introduced a probabilistic representation method based on VAE and semi-supervised learning for the inverse design of artificial metasurfaces.^37^ By representing the metamaterial design problem as a generative process through and sampling in the latent space, they generated structures that meet specific optical responses. This approach addressed the many-to-one mapping problem in inverse design and improved model performance by incorporating semi-supervised learning with unlabeled data.

In summary, the advantages of VAE methods lie in their strong generative capability and modeling ability for complex structures, enabling the rapid generation of metasurface designs that meet specific functional requirements. Compared to traditional optimization methods, VAEs can explore a broader design space, enhancing design efficiency and innovation. This makes VAE a cutting-edge and effective tool in metasurface design.

### Generative adversarial network for inverse design

#### GAN

GAN is a powerful DL model used in metasurface design for generating and optimizing structures with complex electromagnetic properties. GAN consists of two components: a generator and a discriminator, which engage in adversarial training. The generator creates new structures, while the discriminator distinguishes between generated structures and real designs.^167^ In metasurface design, GAN can learn implicit design rules from existing data and generate new metasurface structures that meet specific electromagnetic performance requirements.

#### Application

GAN is a deep generative model built from a generator and a discriminator. The generator is trained adversarially to create samples that ideally cannot be distinguished from the training dataset distribution. Through the interplay between the generator and discriminator, GAN can produce high-quality structures that meet design requirements. Due to their ability to quickly generate large numbers of nanostructures, GAN has been used to design and optimize dielectric and metallic metasurfaces in a randomized manner.^39^

**Figure 16:**
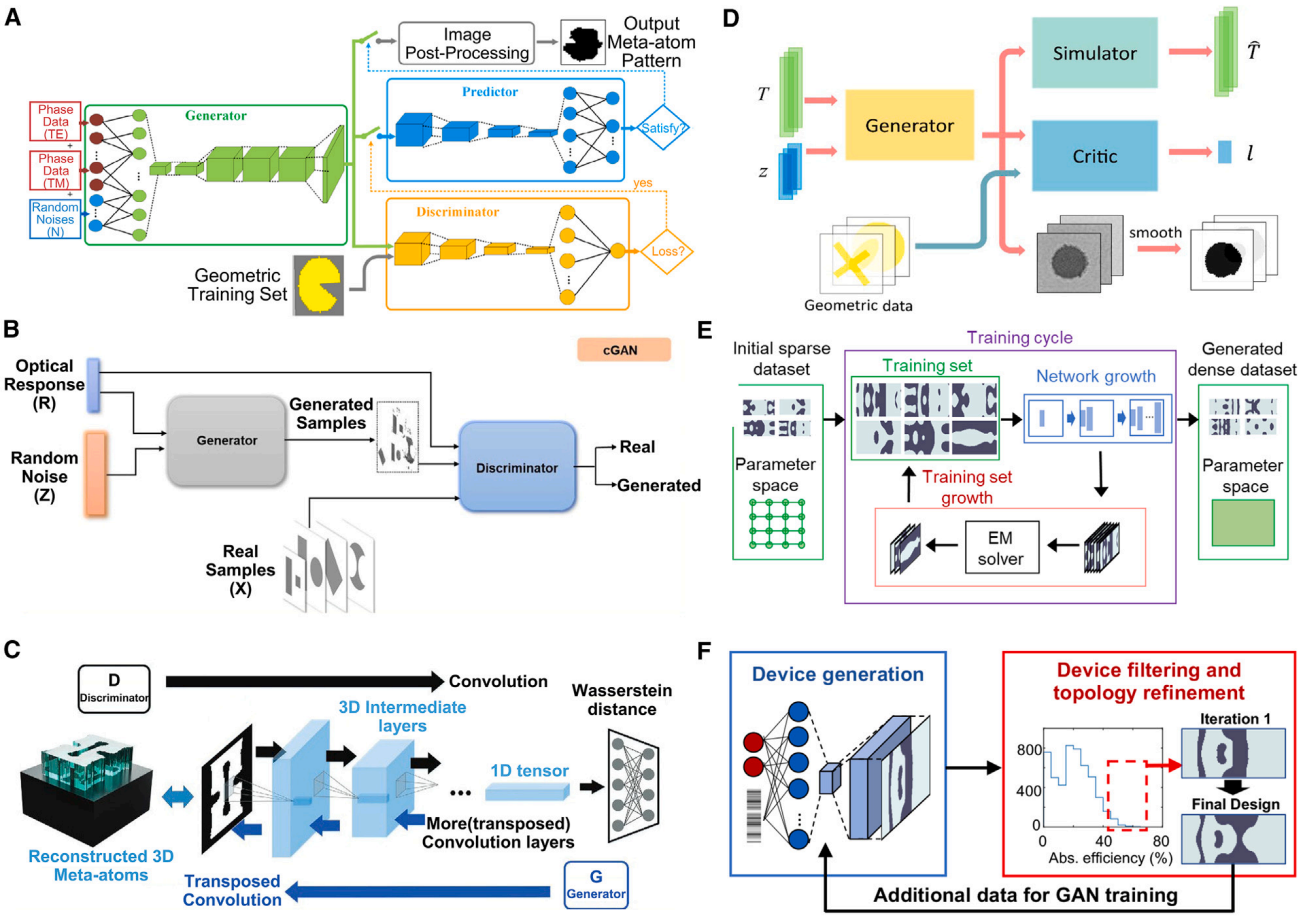
GAN adopted for the inverse design of metasurface (A) Basic architecture of a VAE implemented in the framework. Reproduced with permission, from Liu et al. Copyright 2020,^166^ IEEE. (B) cGAN model architecture to generate structural designs. Reproduced with permission, from Mall et al. Copyright 2020,^43^ Springer Nature. (C) Flow diagram of the generator and discriminator. Reproduced with permission, from An et al. Copyright 2021,^170^ Wiley Online Library. (D) Architecture of the proposed network for AI-based optical design. Reproduced with permission, from Liu et al. Copyright 2018,^171^ American Chemical Society. (E) Overview of GANs based on progressively growing network architectures and training sets. Reproduced with permission, from Wen et al. Copyright 2020,^172^ American Chemical Society. (F) Schematic of metasurface inverse design based on device generation from a trained generative neural network. Reproduced with permission, from Jiang et al. Copyright 2019,^41^ American Chemical Society.

As shown in Figure 16, Xia et al. introduced a deep UN++ GAN for the design and characterization of mixed-function metasurfaces with free modes. The generator utilized a deepened Unet++ structure, and the loss function was restructured to enhance performance. This modification significantly improved the model’s prediction accuracy, with the average accuracy of the two models reaching 96.04% and 94.95%, respectively.^168^ Liu et al. proposed a dual-path network (DPN)-GAN to address the inefficiencies of traditional trial-and-error methods in the inverse design of surface element modes for wideband radar cross-section reduction (RCSR) coding elements. Simulation results demonstrated that the accuracy of 300 test datasets exceeded 97%, underscoring the high predictive capability of the network.^169^

An et al. utilized GAN to design multifunctional metasurfaces.^170^ By combining conditional GAN and Wasserstein GAN (WGAN), they effectively generated free-form all-dielectric metasurfaces meeting multifunctional design goals. Wen et al. proposed a robust free-form metasurface design method based on Progressive Growing GANs (PGGAN).^172^ By introducing a progressive network architecture and self-attention mechanisms, they improved the training process of GAN to generate efficient and robust free-form metasurface structures. Wang et al. used GAN to design ultra-broadband anisotropic metasurfaces.^40^ Through a conditional deep convolutional GAN model, they automatically generated metasurface patterns meeting target phase reflection characteristics by using target reflection spectra as input. Mall et al. employed a GAN-based cyclic DL framework for the inverse design of nanophotonic metasurfaces.^43^ They continuously optimized and generated new structural designs using GAN during the design process. Jiang et al. introduced a GAN-based method for designing free-form diffractive gratings.^41^ By learning geometric features from topology-optimized grating images, GAN generated candidate grating designs that met specific operational parameters, such as wavelength and deflection angle. Liu et al. proposed an inverse design method using GAN for metasurface structures with specific optical properties.^171^ By constructing a DLN architecture that includes a generator, simulator, and critic, they generated metasurface patterns under custom spectral inputs. This approach effectively avoided local optima and identified multiple design solutions with different topologies in a shorter time.

In conclusion, GANs demonstrate exceptional generative capabilities, facilitating the creation of diverse and high-performance metasurface structures within the design space while preserving innovation and practicality. When integrated with traditional optimization methods, GAN can expedite the design process and identify optimal solutions for complex and diverse design tasks, fostering automation and driving high-performance development in metasurface devices.

### Fusion method for inverse design

Integrating various neural network methods can significantly enhance efficiency and innovation in metasurface structural design. Combining different network models, such as CNNs, GANs, VAEs, and RNNs, leverages the strength of each approach. CNNs excel at extracting features from images or structures to optimize geometric parameters, while DNNs handle complex, high-dimensional nonlinear mappings to establish precise relationships between input parameters and performance metrics. GANs generate diverse, high-quality structural designs, VAEs explore latent spaces to discover innovative combinations, and RNNs are adept at processing sequential or related data to optimize performance in dynamic environments. The combined use of these networks enhances design diversity and accelerates structural optimization, achieving multifunctional metasurface designs that meet complex performance requirements.

#### Application

Wang et al. proposed a DL-based space-time encoding digital metasurface component design method that combines CNN, LSTM networks, and DNN into a hybrid algorithm (CLD).^173^ The CLD hybrid algorithm efficiently implements state recognition and mapping for metasurface components, significantly reducing simulation time. Sajedian et al. introduced an image-processing method that combines CNN and RNN to predict the optical properties of metasurface structures.^174^ By generating simulation data of random structures, the model learns spatial information from structural images and uses RNN to predict absorption spectra.

**Figure 17:**
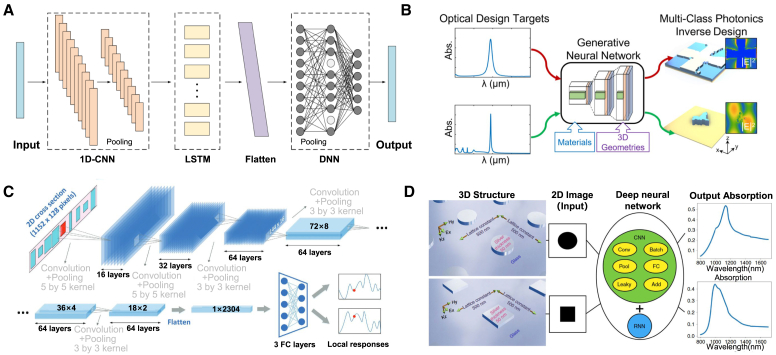
Fusion algorithm adopted for the inverse design of metasurface (A) Architecture of the hybrid CLD model for STCDME state recognition and mapping. Reproduced with permission, from Wang et al. Copyright 2024,^173^ IEEE. (B) Schematic of the cDCGAN training and design process. Reproduced with permission, from Yeung et al. Copyright 2021,^175^ American Chemical Society. (C) 2D cross-section containing the target meta-atom (in red) and its neighbors (in cyan) were processed through six consecutive convolution and pooling layers. Reproduced with permission, from An et al. Copyright 2022,^42^ Wiley Online Library. (D) Schematic of the process, from the 3D structure to the absorption curve output. Reproduced with permission, from Sajedian et al. Copyright 2019,^177^ Springer Nature.

As shown in Figure 17, An et al. employed a deep CNN-based method to predict mutual coupling effects between unit cells in metasurfaces.^42^ This approach enables fast prediction of phase and amplitude responses for optimizing metasurface devices like beam deflectors and metal lenses. Christopher Yeung et al. developed a global inverse design framework based on generative DL, introducing a conditional deep convolutional GAN (cDCGAN).^175^ This framework generates various photonic structures with specific absorption spectra using colored images to encode material and structural parameters. Han et al. proposed an inverse design method combining DNN and CNN for high-degree-of-freedom optical filters.^176^ Their study achieved rapid and accurate design of metasurface structures in the visible light spectrum using generative models and contrast vectors. Sajedian et al. also introduced a deep-learning model combining CNN and RNN to predict the optical properties of plasmonic structures, especially absorption spectra.^177^ By converting 3D structures into 2D images for processing, this approach avoids the high computational costs of traditional numerical simulations and enables accurate optical performance predictions in a short time.

In summary, combining multiple neural network approaches for metasurface structural design maximizes the advantages of each model. This integration enables a multi-objective design that meets complex functional requirements. However, there are challenges, including the complexity of training and tuning, potential difficulties in hyperparameter adjustment, and the black-box nature of combined models, which can limit interpretability and control. Future developments in metasurface design will benefit from increased computational power and algorithm optimization, focusing on improving network integration efficiency, and may trend toward combining physical simulations with DL for better interpretability and control.

## Conclusion and outlooks

This paper discusses methods of metasurface design based on AI. It classifies current design methods into forward and reverse design according to the mapping relationship between structural parameters and optical response. This study investigated intelligent algorithms based on forward design and various network model structures based on reverse design. The development and application of metasurface structure design and its future are comprehensively summarized in this paper. Rapid advances in AI are redefining the paradigm of metasurface design. Traditional design methods rely on repeated physical modeling and numerical simulation, which is time-consuming and labor-intensive. It is difficult for them to meet complex multifunctional requirements. Moreover, by optimizing the metasurface structure, AI can facilitate more efficient light energy management and electromagnetic energy transmission, thereby supporting the development of low-energy devices and green technologies.^178^^,^^179^ AI, especially the combination of DL and optimization, has injected unprecedented flexibility and efficiency into this field. AI can significantly reduce human intervention by automating end-to-end processes from requirement input to design output. For example, a design framework based on GANs can generate high-quality metasurface structures directly from the target performance, significantly shortening the design cycle. Reinforcement learning (RL) can dynamically adjust design strategies based on real-time feedback, enabling AI models to improve during design.^180^ Combining the fast prediction capabilities of DL with the global search capabilities of optimization algorithms, AI handles complex multi-constraint design problems in real time, pushing the design paradigm from relying on domain expert experience to being data-centric and revealing underlying patterns in complex design problems through large-scale data mining and pattern learning. AI plays a role in electromagnetic performance optimization and can work with manufacturing optimization and material design to achieve a true sense of integrated multidisciplinary optimization.

An innovative technology, the design of metasurface structures has shown great potential in many fields, such as optics, electronics, and communication. We foresee that future research on metasurfaces and intelligent design metasurfaces focusing on reconstructing traditional sampling theory and exploring new forms of modulation that can be used in next-generation communication systems by deeply integrating digital information with electromagnetic fields, especially in the time, frequency, airspace, and polarization domains. Since most current work on metasurfaces is in a single frequency band, more working frequency bands will also be an important direction of future research. With the development of DL, RL methods and quantum machine learning methods have been developed to accelerate the learning process. RL is an iterative machine learning method that learns the direction of the current update through interaction with the environment.^181^ It effectively explores a given optimization space to achieve the design goal in the shortest possible time. By changing the material, adjusting the cell size, and using the simulation results as data input, an RL network learns the update operation under different conditions and accurately predicts the next update.^182^ At each step, the expected update strategy is generated. RL is more realistic and suitable for multiband and multi-objective transfer learning than traditional machine learning methods that predict results based directly on raw data. Quantum machine learning leverages the unique quantum superposition and parallelism of quantum computing to achieve efficient data processing and exponential data storage.^183^ Also, using quantum superposition, quantum entanglement, and other characteristics to accelerate the classical machine learning algorithm can get faster, more efficient, and more accurate predictions.^184^ Using quantum characteristics can also solve some high-dimensional data problems that are difficult to deal with using classical machine learning.^185^ Moreover, most quantum machine learning algorithms perform matrix operations on high-dimensional spaces, which is similar to the mathematical properties of quantum computing. Therefore, quantum machine learning has very high research value for complex metasurface structures and complex tasks with very large amounts of data. In the field of intelligent metasurfaces, there are still many possible avenues of research and improved research methods worth exploring.

## Acknowledgments

This work was supported in part by the 10.13039/501100001809National Natural Science Foundation of China under Grant 62004166, in part by the 10.13039/501100021171Guangdong Basic and Applied Basic Research Fund 2024A1515012388, in part by the Shaanxi Science Fund for Distinguished Young Scholars under Grant 2022JC-49, in part by 10.13039/501100004731Natural Science Foundation of Zhejiang Province LY23F040002, in part by Natural Science Foundation of Ningbo 202003N4062 and in part by Aeronautical Science Foundation of China 20230008053003.

## Author contributions

G.T.Y.: Conceptualization, writing-original draft. Q.X.X.: Conceptualization and review. Z.L.Z.: Conceptualization and editing. Z.Y.: Editing and review. X.X.W.: Conceptualization and review. Q.B.L.: Supervision and fund acquisition.

## Declaration of interests

The authors declare no competing interests.
