# Supplementary material for: Exploring AI in metasurface structures with forward and inverse design
Source: iScience. 2025 Feb 15;28(3):111995. doi: 10.1016/j.isci.2025.111995 (PMC11914293; doi:10.1016/j.isci.2025.111995)
Supplement: Document S1. Tables S1–S4 [file mmc1.pdf]

iScience, Volume *28*

Supplemental information

Exploring AI in metasurface structures

with forward and inverse design

Guantai Yang, Qingxiong Xiao, Zhilin Zhang, Zhe Yu, Xiaoxu Wang, and Qianbo Lu

Supplemental Tables

Algorithm	Accuracy	Efficiency	Convergency	Reference
GA	Good	Average	Fair	Yu et al.[1], Liu et al.[2], Xu et al.[3], Jiang et al.[4]
PSO	Excellent	Fair	Average	Kim et al.[5], Ong et al.[6], Mahmoud et al.[7], Zhang et al.[8]
ACO	Average	Poor	Good	Whiting et al.[9], Lewis et al.[10], Zhu et al.[11], Socha et al.[12]
DBS	Fair	Excellent	Average	Liu et al.[13], Ma et al.[14], Lee et al.[15], Jiang et al.[60]
AM	Good	Average	Poor	Oh et al.[16], Mansouree et al.[17], Zhang et al.[18], Zhou et al.[19]
LST	Poor	Good	Average	Guo et al.[20], Dong et al.[21], Emoto et al.[22], Noguchi et al.[23]
DTO	Average	Average	Good	Hammond et al.[24], Lin et al.[25], Yang et al.[26], Phan et al.[27]
Fusion method	Average	Good	Excellent	Wang et al.[28], Zhu et al.[29], Wu et al.[30], Yu et al.[31]

Table S1. Performance of forward design algorithm. Performance ratings are divided into five registrations, which are Excellent, Good, Average, Fair, and Poor.

Algorithm	Accuracy	Efficiency	Convergency	Reference
CNN	Good	Average	Poor	Shan et al.[32], Zhang et al.[33], Zhu et al.[34], Lin et al.[35]
DNN	Excellent	Poor	Average	Tahersima et al.[36], An et al.[37], Nadell et al.[38], Liu et al.[39]
RNN	Average	Average	Fair	Sherstinsky et al.[40], Hochreiter et al.[41], Mao et al.[42], Zhang et al.[43]
VAE	Fair	Good	Average	Tang et al.[44], Chen et al.[45], Wei et al.[46], Naseri et al.[47]
GAN	Good	Fair	Average	Jiang et al.[48], An et al.[49], Liu et al.[50], Wen et al.[51]
Fusion method	Good	Average	Excellent	Han et al.[52], Wang et al.[53], Tanri-over et al.[54], Yeung et al.[55]

Table S2. Performance of inverse design algorithm. Performance ratings are divided into five registrations, which are Excellent, Good, Average, Fair, and Poor.

Algorithm	Parameter Space	Accuracy	Convergence Speed	Reference
GA	Average	Good	Poor	Yu et al.[1], Liu et al.[2], Xu et al.[3], Jiang et al.[4]
PSO	Good	Excellent	Average	Kim et al.[5], Ong et al.[6], Mahmoud et al.[7], Zhang et al.[8]
ACO	Excellent	Poor	Fair	Whiting et al.[9], Lewis et al.[10], Zhu et al.[11], Socha et al.[12]
DBS	Fair	Average	Good	Liu et al.[13], Ma et al.[14], Lee et al.[15], Jiang et al.[60]
AM	Average	Fair	Average	Oh et al.[16], Mansouree et al.[17], Zhang et al.[18], Zhou et al.[19]
LST	Poor	Average	Average	Guo et al.[20], Dong et al.[21], Emoto et al.[22], Noguchi et al.[23]
DTO	Average	Average	Excellent	Hammond et al.[24], Lin et al.[25], Yang et al.[26], Phan et al.[27]
Fusion method	Good	Good	Excellent	Wang et al.[28], Zhu et al.[29], Wu et al.[30], Yu et al.[31]

Table S3. Computing resources required for forward design. The computational resources required for design are divided into five levels: Excellent, Good, Average, Fair, and Poor.

Algorithm	Params	FLOPs	Accuracy	Reference
CNN	Good	Average	Fair	Shan et al.[32], Zhang et al.[33], Zhu et al.[34], Lin et al.[35]
DNN	Excellent	Fair	Poor	Tahersima et al.[36], An et al.[37], Nadell et al.[38], Liu et al.[39]
RNN	Poor	Good	Average	Sherstinsky et al.[40], Hochreiter et al.[41], Mao et al.[42], Zhang et al.[43]
VAE	Average	Poor	Good	Tang et al.[44], Chen et al.[45], Wei et al.[46], Naseri et al.[47]
GAN	Good	Average	Excellent	Jiang et al.[48], An et al.[49], Liu et al.[50], Wen et al.[51]
Fusion method	Good	Excellent	Good	Han et al.[52], Wang et al.[53], Tanriover et al.[54], Yeung et al.[55]

Table S4. Computing resources required for inverse design. The params refers to the computational space complexity, while the ELOPs refers to the computational time complexity. The computational resources required for design are divided into five levels: Excellent, Good, Average, Fair, and Poor.

References

1. Yu, Z., Chen, C., Chen, W., Zhang, X., and Zhang, X. (2024). Cosecant Squared Beam-Forming by Metasurface With Complex-Amplitude Controls for Antenna Array. IEEE Antennas and Wireless Propagation Letters. https://doi.org/10.1109/LAWP.2024.3506581.

2. Liu, S., Ye, Z., Tan, R., Han, M., Zhuang, H., and Chen, P. (2024). Genetic algorithm-enabled on-demand co-design of optically transparent metamaterial for multispectral stealth applications. Optics Express *32*, 30969–30981. https://doi.org/10.1364/OE.529553.

3. Xu, Z.-L., Wang, D.-F., Shi, Y.-F., Qian, Z.-H., Assouar, B., and Chuang, K.-C. (2023). Arbitrary wavefront modulation utilizing an aperiodic elastic metasurface. INTERNATIONAL JOURNAL OF MECHANICAL SCIENCES *255*. MAY 2023. https://doi.org/10.1016/j.ijmecsci.2023.108460.

4. Jiang, X., Yuan, H., Chen, D., Zhang, Z., Du, T., Ma, H., and Yang, J. (2021). Metasurface Based on Inverse Design for Maximizing Solar Spectral Absorption. ADVANCED OPTICAL MATERIALS *9*. JUN 2021. https://doi.org/10.1002/adom.202100575.

5. Kim, J., Kim, J.-Y., Yoon, J., Yoon, H., Park, H.-H., and Kurt, H. (2022). Experimental demonstration of inverse-designed silicon integrated photonic power splitters. NANOPHOTONICS *11*. SEP 2022, 4581–4589. https://doi.org/10.1515/nanoph-2022-0443.

6. Ong, J. R., Chu, H. S., Chen, V. H., Zhu, A. Y., and Genevet, P. (2017). Freestanding dielectric nanohole array metasurface for mid-infrared wavelength applications. OPTICS LETTERS *42*. Article, 2639–2642. https://doi.org/10.1364/OL.42.002639.

7. Mahmoud, K. R., Hussein, M., Hameed, M. F. O., and Obayya, S. S. A. (2017). Super directive Yagi-Uda nanoantennas with an ellipsoid reflector for optimal radiation emission. JOURNAL OF THE OPTICAL SOCIETY OF AMERICA B-OPTICAL PHYSICS *34*. Article, 2041–2049. https://doi.org/10.1364/JOSAB.34.002041.

8. Zhang, J., Zhu, H., Ghosh, P., and Li, Q. (2024a). “A visible transparent infrared microwave compatible stealth metasurface with low infrared emissivity”. *Journal of Physics: Conference Series*. Vol. 2873. IOP Publishing, 012043. https://doi.org/10.1088/1742-6596/2873/1/012043.

9. Whiting, E. B., Campbell, S. D., Werner, D. H., Werner, P. L., and IEEE (2020). *Design Methods for 3D Membrane Projection Lithography Metasurface Unit Cells*. Proceedings Paper. https://doi.org/10.1109/metamaterials49557.2020.9285106.

10. Lewis, A., Weis, G., Randall, M., Galehdar, A., Thiel, D., and IEEE (2009). *Optimising Efficiency and Gain of Small Meander Line RFID Antennas using Ant Colony System*. Proceedings Paper. https://doi.org/10.1109/CEC.2009.4983118.

11. Zhu, D. Z., Whiting, E. B., Campbell, S. D., Burckel, D. B., and Werner, D. H. (2019). Optimal High Efficiency 3D Plasmonic Metasurface Elements Revealed by Lazy Ants. ACS PHOTONICS *6*. Article, 2741–2748. https://doi.org/10.1021/acsphotonics.9b00717.

12. Zhu, D. Z., Werner, P. L., and Werner, D. H. (2017). Design and Optimization of 3-D Frequency-Selective Surfaces Based on a Multiobjective Lazy Ant Colony Optimiza-

tion Algorithm. IEEE TRANSACTIONS ON ANTENNAS AND PROPAGATION *65*. Article, 7137–7149. https://doi.org/10.1109/TAP.2017.2766660.

13. Liu, B., Wan, Y., and Liu, Y. (2024). Ultracompact Polarization-Insensitive Waveguide Crossing Based on Dielectric Metasurface. IEEE PHOTONICS TECHNOLOGY LETTERS *36*. Article, 39–42. https://doi.org/10.1109/LPT.2023.3337360.

14. Ma, H., Du, T., Jiang, X., Zhang, Z., He, X., Chen, H., Yu, Y., Zhang, Z., Han, Y., Yang, J., et al. (2023). Inverse-designed ultra-compact multi-channel and multi-mode waveguide crossings. OPTICS EXPRESS *31*. Article, 29235–29244. https://doi.org/10.1364/OE.500327.

15. Lee, J., Jia, W., Sensale-Rodriguez, B., Walling, J. S., and IEEE (2023). *Pixelated RF: Random Metasurface Based Electromagnetic Filters*. Proceedings Paper. https://doi.org/10.1109/NEWCAS57931.2023.10198065.

16. Oh, J., Yang, J., Marra, L., Dorrah, A. H., Palmieri, A., Dainese, P., and Capasso, F. (2024). Metasurfaces for Free-Space Coupling to Multicore Fibers. JOURNAL OF LIGHTWAVE TECHNOLOGY *42*. Article, 2385–2396. https://doi.org/10.1109/JLT.2023.3335334.

17. Ma, H., Bao, G., Lai, J., and Lin, J. (2024). Inverse design of a grating metasurface for enhancing spontaneous emission through hyperbolic metamaterials. JOURNAL OF THE OPTICAL SOCIETY OF AMERICA B-OPTICAL PHYSICS *41*. Article, A79–A85. https://doi.org/10.1364/JOSAB.497519.

18. Zhang, D., Liu, Z., Yang, X., and Xiao, J. J. (2022). Inverse Design of Multifunctional Metasurface Based on Multipole Decomposition and the Adjoint Method. ACS PHOTONICS *9*. NOV 2022, 3899–3905. https://doi.org/10.1021/acsphotonics.2c01187.

19. Zhou, M., Liu, D., Belling, S. W., Cheng, H., Kats, M. A., Fan, S., Povinelli, M. L., and Yu, Z. (2021). Inverse Design of Metasurfaces Based on Coupled-Mode Theory and Adjoint Optimization. ACS PHOTONICS *8*. JUL 2021, 2265–2273. https://doi.org/10.1021/acsphotonics.1c00100.

20. Guo, J. and Cheng, H. (2024). Acoustic metasurface development for transmitted wavefront manipulation using a level set-based topology optimization approach. JOURNAL OF SOUND AND VIBRATION *579*. MAR 2024. https://doi.org/10.1016/j.jsv.2024.118382.

21. Dong, L., Kong, W., Wang, C., Pu, M., Liu, X., Luo, Y., and Luo, X. (2022). Quasi-continuous metasurface for high-efficiency beam deflection based on multi-objective level-set optimization. OPTICAL MATERIALS EXPRESS *12*. Article, 3667–3678. https://doi.org/10.1364/OME.470765.

22. Emoto, H., Noguchi, Y., and Yamada, T. (2023). Acoustic metasurfaces designed via topology optimization for regional sound insulation. JOURNAL OF SOUND AND VIBRATION *567*. JUL 2023. https://doi.org/10.1016/j.jsv.2023.117939.

23. Noguchi, Y. and Yamada, T. (2021). Topology optimization of acoustic metasurfaces by using a two-scale homogenization method. APPLIED MATHEMATICAL MODELLING *98*. JUN 2021, 465–497. https://doi.org/10.1016/j.apm.2021.05.005.

24. Hammond, A. M., Oskooi, A., Johnson, S. G., and Ralph, S. E. (2021). Photonic topology optimization with semiconductor-foundry design-rule constraints. OPTICS EXPRESS *29*. Article, 23916–23938. https://doi.org/10.1364/OE.431188.

25. Lin, Z., Liu, V., Pestourie, R., and Johnson, S. G. (2019). Topology optimization of freeform large-area metasurfaces. OPTICS EXPRESS *27*. Article, 15765–15775. https://doi.org/10.1364/OE.27.015765.

26. Yang, J. and Fan, J. A. (2017). Analysis of material selection on dielectric metasurface performance. OPTICS EXPRESS *25*. Article, 23899–23909. https://doi.org/10.1364/OE.25.023899.

27. Phan, T., Sell, D., Wang, E. W., Doshay, S., Edee, K., Yang, J., and Fan, J. A. (2019). High-efficiency, large-area, topology-optimized metasurfaces. LIGHT-SCIENCE & APPLICATIONS *8*. 48. https://doi.org/10.1038/s41377-019-0159-5.

28. Wang, L., Dong, J., Zhang, W., Zheng, C., and Liu, L. (2024a). Inverse design for laser-compatible infrared camouflage metasurface enabled by physics-driven neural network and genetic algorithm. OPTICAL MATERIALS *153*. JUN 2024. https://doi.org/10.1016/j.optmat.2024.115639.

29. Zhu, R., Qiu, T., Wang, J., Sui, S., Li, Y., Feng, M., Ma, H., and Qu, S. (2020). Multiplexing the aperture of a metasurface: inverse design via deep-learning-forward genetic algorithm. JOURNAL OF PHYSICS D-APPLIED PHYSICS *53*. 455002. https://doi.org/10.1088/1361-6463/aba64f.

30. Wu, G., Si, L., Xu, H., Niu, R., Zhuang, Y., Zhuang, H., Sun, H., and Ding, J. (2022). Phase-to-pattern inverse design for a fast realization of a functional metasurface by combining a deep neural network and a genetic algorithm. OPTICS EXPRESS *30*. Article, 45612–45623. https://doi.org/10.1364/OE.478084.

31. Yu, R., Liu, Y., and Zhu, L. (2022). Inverse design of high degree of freedom meta-atoms based on machine learning and genetic algorithm methods. OPTICS EXPRESS *30*. Article, 35776–35791. https://doi.org/10.1364/OE.472280.

32. Shan, T., Pan, X., Li, M., Xu, S., and Yang, F. (2020). Coding Programmable Metasurfaces Based on Deep Learning Techniques. IEEE JOURNAL ON EMERGING AND SELECTED TOPICS IN CIRCUITS AND SYSTEMS *10*. Article, 114–125. https://doi.org/10.1109/JETCAS.2020.2972764.

33. Zhang, Q., Liu, C., Wan, X., Zhang, L., Liu, S., Yang, Y., and Cui, T. J. (2019). Machine-Learning Designs of Anisotropic Digital Coding Metasurfaces. ADVANCED THEORY AND SIMULATIONS *2*. 1800132. https://doi.org/10.1002/adts.201800132.

34. Zhu, L., Zhang, C., Guo, J., Dong, L., and Gong, J. (2022). Deep learning for electromagnetically induced transparency (EIT) metasurface optimization design. JOURNAL OF PHYSICS D-APPLIED PHYSICS *55*. 315001. https://doi.org/10.1088/1361-6463/ac670f.

35. Lin, R., Zhai, Y., Xiong, C., and Li, X. (2020). Inverse design of plasmonic metasurfaces by convolutional neural network. OPTICS LETTERS *45*. Article, 1362–1365. https://doi.org/10.1364/OL.387404.

36. Tahersima, M. H., Kojima, K., Koike-Akino, T., Jha, D., Wang, B., Lin, C., and Parsons, K. (2019). Deep Neural Network Inverse Design of Integrated Photonic Power Splitters. SCIENTIFIC REPORTS *9*. 1368. https://doi.org/10.1038/s41598-018-37952-2.

37. An, S., Zheng, B., Julian, M., Williams, C., Tang, H., Gu, T., Zhang, H., Kim, H. J., and Hu, J. (2022). Deep neural network enabled active metasurface embedded design. NANOPHOTONICS *11*. JUN 2022, 4149–4158. https://doi.org/10.1515/nanoph-2022-0152.

38. Nadell, C. C., Huang, B., Malof, J. M., and Padilla, W. J. (2019). Deep learning for accelerated all-dielectric metasurface design. OPTICS EXPRESS *27*. Article, 27523–27535. https://doi.org/10.1364/OE.27.027523.

39. Liu, D., Tan, Y., Khoram, E., and Yu, Z. (2018a). Training Deep Neural Networks for the Inverse Design of Nanophotonic Structures. ACS PHOTONICS *5*. Article, 1365–1369. https://doi.org/10.1021/acsphotonics.7b01377.

40. Sherstinsky, A. (2020). Fundamentals of Recurrent Neural Network (RNN) and Long Short-Term Memory (LSTM) network. PHYSICA D-NONLINEAR PHENOMENA *404*. 132306. https://doi.org/10.1016/j.physd.2019.132306.

41. Hochreiter, S. and Schmidhuber, J. (1997). Long short-term memory. NEURAL COMPUTATION *9*. Article, 1735–1780. https://doi.org/10.1162/neco.1997.9.8.1735.

42. Mao, B., Chang, H., Xing, X., Zhang, Q., Zou, D., Liu, Y., Yao, J., Bi, H., and Wu, L. (2024). Designing ultra-broadband terahertz polarization converters based on the transformer model. OPTICS COMMUNICATIONS *559*. MAR 2024. https://doi.org/10.1016/j.optcom.2024.130434.

43. Zhang, J., Qian, C., You, G., Wang, T., Saifullah, Y., Abdi-Ghaleh, R., and Chen, H. (2024b). Harnessing the Missing Spectral Correlation for Metasurface Inverse Design. ADVANCED SCIENCE. JUL 2024. https://doi.org/10.1002/advs.202308807.

44. Tang, Y., Kojima, K., Koike-Akino, T., Wang, Y., Wu, P., Tahersima, M., Jha, D., Parsons, K., and Qi, M. (2020). “Generative deep learning model for a multi-level nano-optic broadband power splitter”. *Optical Fiber Communication Conference*. Optica Publishing Group, Th1A–1. https://doi.org/10.1364/OFC.2020.Th1A.1.

45. Chen, J., Qian, C., Zhang, J., Jia, Y., and Chen, H. (2023). Correlating metasurface spectra with a generation-elimination framework. NATURE COMMUNICATIONS *14*. 4872. https://doi.org/10.1038/s41467-023-40619-w.

46. Wei, Z., Zhou, Z., Wang, P., Ren, J., Yin, Y., Pedersen, G. F., and Shen, M. (2022). Equivalent Circuit Theory-Assisted Deep Learning for Accelerated Generative Design of Metasurfaces. IEEE TRANSACTIONS ON ANTENNAS AND PROPAGATION *70*. Article, 5120–5129. https://doi.org/10.1109/TAP.2022.3152592.

47. Naseri, P. and Hum Sean, V. (2021). A Generative Machine Learning-Based Approach for Inverse Design of Multilayer Metasurfaces. IEEE TRANSACTIONS ON ANTENNAS AND PROPAGATION *69*. Article, 5725–5739. https://doi.org/10.1109/TAP.2021.3060142.

48. Jiang, J., Sell, D., Hoyer, S., Hickey, J., Yang, J., and Fan, J. A. (2019). Free-Form Diffractive Metagrating Design Based on Generative Adversarial Networks. ACS NANO *13*. Article, 8872–8878. https://doi.org/10.1021/acsnano.9b02371.

49. An, S., Zheng, B., Tang, H., Shalaginov, M. Y., Zhou, L., Li, H., Kang, M., Richardson, K. A., Gu, T., Hu, J., et al. (2021). Multifunctional Metasurface Design with a Generative Adversarial Network. ADVANCED OPTICAL MATERIALS *9*. JAN 2021. https://doi.org/10.1002/adom.202001433.

50. Liu, Z., Zhu, D., Rodrigues, S. P., Lee, K.-T., and Cai, W. (2018b). Generative Model for the Inverse Design of Metasurfaces. NANO LETTERS *18*. Article, 6570–6576. https://doi.org/10.1021/acs.nanolett.8b03171.

51. Wen, F., Jiang, J., and Fan, J. A. (2020). Robust Freeform Metasurface Design Based on Progressively Growing Generative Networks. ACS PHOTONICS *7*. Article, 2098–2104. https://doi.org/10.1021/acsphotonics.0c00539.

52. Han, X., Fan, Z., Liu, Z., Li, C., and Guo, L. J. (2021). Inverse design of metasurface optical filters using deep neural network with high degrees of freedom. INFOMAT *3*. JUN 2020, 432–442. https://doi.org/10.1002/inf2.12116.

53. Wang, P., Li, Z., Wei, Z., Wu, T., Luo, C., Jiang, W., Hong, T., Pedersen, G. F., and Shen, M. (2024b). Space-Time-Coding Digital Metasurface Element Design Based on State Recognition and Mapping Methods With CNN-LSTM-DNN. IEEE TRANSACTIONS ON ANTENNAS AND PROPAGATION *72*. Article, 4962–4975. https://doi.org/10.1109/TAP.2024.3349778.

54. Tanriover, I., Lee, D., Chen, W., and Aydin, K. (2023). Deep Generative Modeling and Inverse Design of Manufacturable Free-Form Dielectric Metasurfaces. ACS PHOTONICS *10*. SEP 2022, 875–883. https://doi.org/10.1021/acsphotonics.2c01006.

55. Yeung, C., Tsai, R., Pham, B., King, B., Kawagoe, Y., Ho, D., Liang, J., Knight, M. W., and Raman, A. P. (2021). Global Inverse Design across Multiple Photonic Structure Classes Using Generative Deep Learning. ADVANCED OPTICAL MATERIALS *9*. JUL 2021. https://doi.org/10.1002/adom.202100548.
